# Synthesis, Characterization, and Self-Assembly Behavior of Block Copolymers of N-Vinyl Pyrrolidone with n-Alkyl Methacrylates

**DOI:** 10.3390/polym17081122

**Published:** 2025-04-21

**Authors:** Nikoletta Roka, Marinos Pitsikalis

**Affiliations:** Industrial Chemistry Laboratory, Department of Chemistry, National and Kapodistrian University of Athens, Panepistimiopolis Zografou, 15771 Athens, Greece; nikolettaroka@yahoo.gr

**Keywords:** poly(N-vinyl pyrrolidone), poly(n-alkyl methacrylates), block copolymers, micelles, encapsulation of drugs

## Abstract

Novel amphiphilic block copolymers of N-vinyl pyrrolidone (NVP) and either n-hexyl methacrylate (HMA, PNVP-*b*-PHMA) or stearyl methacrylate (SMA, PNVP-*b*-PSMA) were prepared by RAFT polymerization techniques and the sequential addition of monomers starting from the polymerization of NVP and using two different Chain Transfer Agents, CTAs. PNVP-*b*-PHMA are amorphous block copolymers containing constituent blocks with both high and low Tg values, whereas PNVP-*b*-PSMA are amorphous–semi-crystalline copolymers. Samples with different molecular weights and compositions were obtained. The copolymers were microphase-separated, but partial mixing was also observed. The presence of the amorphous PNVP block reduced the crystallinity of the PSMA blocks in the PNVP-*b*-PSMA copolymers. The thermal stability of the blocks was influenced by both constituents. The self-assembly behavior in THF, which is a selective solvent for polymethacrylate blocks, and in aqueous solutions, where PNVP was soluble, was examined. Unimolecular or low-aggregation-number micelles were obtained in THF for both types of samples. On the contrary, high-aggregation-number, spherical, and compact micelles were revealed in aqueous solutions. The increase in the steric hindrance of the side ester group of the polymethacrylate chain led to slightly lower degrees of association. The hydrophobic compound curcumin was efficiently encapsulated within the micellar core of the supramolecular structures in aqueous solutions. Micelles with higher aggregation numbers were more efficient in the encapsulation of curcumin. The results of this study were compared with those obtained from other block copolymers based on PNVP.

## 1. Introduction

Poly(N-vinyl pyrrolidone), PNVP, is one of the most important water-soluble polymers with a unique combination of properties including biocompatibility, a lack of toxicity, an ability to form complexes with metal ions, chemical and thermal resistance, an ability to form uniform films, and a high Tg value, which can be reduced upon absorbing humidity [1]. These properties lead to numerous industrial applications, including in the pharmaceutical, biomedical, and cosmetics sectors, where PNVP can be used as a blood plasma substitute, as a tablet binder for the enhancement of drug stability and dissolution, as a solubilizer for suspensions, in wound healing and disinfectant solutions (e.g., PNVP-Iodine), in the dispersion of crystallizing drugs, in tissue engineering (scaffolds), in hydrogels/nanogels, in nanocarriers for drug/gene/protein/peptide delivery, in wound/burn dressings, for the wettability of contact lenses, in dental restoration, and as an antifouling agent [2,3,4,5,6,7,8]. In addition, PNVP can be employed in food products [8,9,10] as well as for environmental protection and for the removal of heavy metals [11] in the synthesis of metal nanoparticles [12,13], etc.

PNVP is an essential material for the development of amphiphilic block copolymers. These are macromolecules composed of two or more chemically distinct polymer segments, typically a hydrophilic (water-attracting) block and a hydrophobic (water-repelling) block [14,15,16,17]. This dual nature allows them to self-assemble into various nanostructures such as micelles, vesicles, and hydrogels, making them highly valuable in biomedical, pharmaceutical, and nanotechnology applications [18,19,20,21,22,23,24]. Numerous linear (diblocks, triblocks, multiblocks) and non-linear (miktoarm stars, grafts, branched polymers) structures have been synthesized, offering tools for numerous applications [25,26,27,28,29,30,31,32,33,34,35,36,37].

Among the various hydrophilic polymers, poly(ethylene oxide), PEO, has been mostly employed for biomedical and pharmaceutical applications. The biocompatibility, low toxicity, commercial availability of samples with various molecular weights and end-groups along with their stealth properties, which prevent the recognition of nanoparticles and uptake by the reticuloendothelial system, are the main advantages of PEO for these applications [38,39,40,41,42]. However, the recent developments of COVID-19 vaccines revealed several drawbacks of PEO as a stealth material [43,44,45,46]. For example, reduced interactions between liposomes and cell membranes were confirmed when liposomes were grafted with PEO, leading to decreased cellular uptake and endosomal escape, thus preventing gene delivery. In addition, the repeated administration of PEO-covered nanoparticles led to immunogenicity implications. PEO was also found to be responsible for mild and even severe allergic reactions in patients. Therefore, there is a need for alternative solutions replacing PEO as the hydrophilic material. In this direction, PNVP has been proven to be an adequate substitute for PEO as a water-soluble polymer. Several studies have pointed to this direction and provided evidence that PNVP is an ideal replacement of PEO [47,48,49,50].

The limiting factor preventing the extended use of PNVP in pharmaceutical applications was, for many years, the difficulties in controlling the polymerization of the NVP monomer. However, recent developments in the Reversible Addition Fragmentation Chain Transfer (RAFT) polymerization technique have offered the opportunity to synthesize well-defined statistical, deblock, and triblock copolymers along with more complex architectures, such as star, graft, and other branched copolymers [51,52]. The ability of these structures to self-assemble in selective solvents has already been demonstrated.

In this work, the synthesis of amphiphilic block copolymers of NVP with n-hexyl-, HMA, and stearyl methacrylate, SMA, is described. Poly(n-hexyl methacrylate), PHMA, is a hydrophobic, water-insoluble polymer derived from the HMA monomer. It is known for its flexibility, low glass transition temperature, Tg, and potential applications in protective coatings, offering flexibility and hydrophobicity, pressure-sensitive adhesives for packaging and medical tapes, biomedical research on drug delivery systems, and bioinert coatings; due to its non-toxic and non-degradable properties and flexible electronics and optoelectronics, it is used as a polymer matrix in organic electronics and flexible displays and in light-sensitive coatings and optical films [53,54,55,56]. On the other hand, poly(stearyl methacrylate), PSMA, is a hydrophobic polymer derived from the SMA monomer, which contains a long-chain C18 stearyl group. This structure gives PSMA unique properties such as side-chain crystallinity, a low glass transition temperature, Tg, hydrophobicity, and self-assembling behavior, making it valuable in hydrophobic coatings for textiles, glass, and metals, biomedical applications and drug delivery systems regarding nanoparticle coatings and the encapsulation of hydrophobic drugs, adhesives, improving the adhesion of hydrophobic films and lubricants, providing low-friction surfaces due to its waxy texture, cosmetics, and personal care products [57,58,59,60].

The synthesis of the block copolymers took place via RAFT polymerization, and the sequential addition of monomers was employed. The polymers were characterized through size-exclusion chromatography, SEC, and NMR spectroscopy. Their thermal properties were studied via Differential Scanning Calorimetry, DSC, and thermogravimetric analysis, TGA. Static, SLS, and dynamic light scattering, DLS, were employed to study the micellization behavior in tetrahydrofuran, THF, which is selective for PHMA and PSMA blocks [61] and in aqueous solutions, where PNVP is soluble. The encapsulation of the hydrophobic compound curcumin was studied by UV spectroscopy.

The main focus of this study has to do with the synthesis of novel amphiphilic block copolymers. PNVP-*b*-PHMA blocks have two amorphous constituents, one with a high Tg and another one with a low Tg value. On the other hand, PNVP-*b*-PSMA copolymers are amorphous–semi-crystalline. The combination of monomers with different reactivities in stabilizing radicals is very challenging in RAFT polymerization. In the past, block copolymers of NVP with benzyl methacrylate, BzMA, [62] and isobornyl methacrylate, IBMA [63], were prepared and their self-assembly properties were studied both in THF and in aqueous solutions. In these studies, emphasis was given on the effect of the macromolecular architecture on the micellization behavior, since the block copolymers were compared with corresponding statistical copolymers. In the present work, the focus is on the nature of the side ester group of the polymethacrylate block in order to elucidate the effect of the hydrophobicity and the flexibility of this side group on the self-organization process in selective solvents. This work is included in the general effort to study the possible replacement of PEO with PNVP as the water-soluble component in amphiphilic block copolymers, considering the problems encountered with the use of PEO in drug delivery systems.

## 2. Materials and Methods

### 2.1. Materials

N-Vinyl pyrrolidone (≥97% FLUKA) stabilized with sodium hydroxide as an inhibitor was dried overnight over calcium hydride and was distilled prior to use. Tetrahydrofuran, THF, was dried over sodium overnight and was distilled just prior to use. Benzene and hexyl methacrylate, HMA (TCI Chemicals, Tokyo, Japan), which contained methyl hydroquinone, were dried over calcium hydride overnight and were then distilled under vacuum prior the polymerization. Stearyl methacrylate, SMA, was dissolved in THF, and the solution was eluted through an inhibitor remover column (Sigma Aldrich, St. Louis, MO, USA). THF was then evaporated and the received SMA monomer was dried in a vacuum oven. Azobisisobutyronitrile, AIBN (98% ACROS), was recrystallized twice from methanol and was then dried under vacuum. The Chain Transfer Agent (CTA), O-ethyl S-(phthalimidylmethyl) xanthate (CTA1), was synthesized following protocols from the literature [64,65].

### 2.2. Synthesis of Phthalimidylmethyl Dithiobenzoate, CTA XM

Phthalimidylmethyl dithiobenzoate, CTA XM, was synthesized according to the following procedure: in a round-bottom flask, 0.045 mols of carbon disulfide, CS_2_, was dissolved in 30 mL of anhydrous THF. A 3 M diethyl ether solution of the Grignard reagent phenyl magnesium bromide (14 mL) was added dropwise at room temperature into the CS_2_ solution over a period of 30 min. The resulting red solution was then heated at 40 °C and 0.04 mols of N-(bromomethyl)phthalimide, dissolved in THF, was added dropwise. The final solution was heated at 50 °C for 12 h. Afterwards, 100 mL of chilled water was added and the pure CTA MX was collected through extraction with CHCl_3_. The collected solution was dried over MgSO_4_. Column chromatography using a mixture of petroleum ether and ethyl acetate (gradient from 8:2 to 7:3) was employed to purify the product. All other reagents and solvents were of commercial grade and were used as received.

### 2.3. Synthesis of PNVP-b-PHMA and PNVP-b-PSMA Block Copolymers via RAFT Polymerization

The synthesis of the block copolymers was accomplished in glass reactors using high-vacuum techniques [66,67], with O–ethyl S–(phthalimidylmethyl) xanthate, CTA1, or phthalimidylmethyl dithiobenzoate, CTA XM, as the CTA, and by the sequential addition of monomers, starting from the polymerization of NVP in all cases. All the PNVP-*b*-PSMA block copolymers and the block copolymers PNVP-*b*-PHMA #1 and #2 were prepared with CTA1, whereas the samples PNVP-*b*-PHMA #3 and #4 were prepared with CTA XM.

As an example, the synthesis of PNVP with final M_n_ = 6.3 × 10^3^ (Table 1, sample #4) with a molar ratio of [NVP]_0_/[CTA MX]_0_/[AIBN]_0_ = 100/1/0.2 involved the following: 5 g of NVP were reacted by employing a mixture of 0.0140 g of CTA XM and 0.0080 g of AIBN in 0.5 mL of benzene. The polymerization mixture was degassed by employing three freeze–thaw pump cycles. The reactor was flame-sealed and placed in a preheated oil-bath at 60 °C for 12 h. The polymerization reaction was terminated by removing the reactor from the oil-bath and cooling the mixture in cold water. The reactor was then opened to the atmosphere. The homopolymer was then precipitated in an excess of diethyl ether. The polymer was redissolved and reprecipitated three times to achieve the complete removal of any residual quantity of unreacted NVP monomers. The sample was then dried overnight in a vacuum oven at 50 °C to remove remaining traces of solvent. The conversions for all homopolymers were near quantitative when CTA1 was employed and about 65% when CTA XM was used.

Copolymerization reactions were allowed to take place in dioxane solutions at 80 °C for 72 h in all glass reactors employing high vacuum. The quantities of the various reagents (macro-CTA PNVP homopolymer, HMA monomer, AIBN, and dioxane) are reported in the Supporting Information Section, SIS (Table S1). The corresponding quantities for the block copolymers with PSMA blocks are also given in the SIS (Table S2). According to the general procedure, three freeze–thaw pump cycles were conducted using the reaction mixture for the elimination of oxygen from the reaction medium. The polymerization procedure was terminated by removing the polymerization flask from the heating bath and cooling the mixture in cold water. The reactor was then opened to expose the mixture to the atmosphere. The product was then precipitated in methanol. The crude polymer was dissolved in THF and reprecipitated in methanol. This procedure took place three times for the efficient removal of any unreacted methacrylate monomers. Subsequently, the polymers were dried overnight in a vacuum oven at 50 °C to remove any residual solvent. The block copolymers PNVP-*b*-PHMA #1 and #4 along with the copolymer PNVP-*b*-PSMA #1 were further purified from the unreacted excess PNVP block by fractionation using chloroform/methanol as the solvent/non-solvent system.

### 2.4. Encapsulation Procedure

Encapsulation studies were conducted preparing different solutions in THF, referring to the block copolymer and the hydrophobic drug curcumin separately. The concentrations for both types of block copolymers, containing either PHMA or PSMA as the hydrophobic block, were close to 5 × 10^−4^ g/mL. The exact concentration values are given in the SIS (Tables S3 and S4). The solutions were allowed to stand overnight for complete dissolution with periodic agitation. After that, the block copolymer solution was split into four separate vials, and a different amount of the curcumin stock solution was added to each one. After efficient mixing, 5 mL of extra-pure water was added to the respective vials. THF was then allowed to evaporate gradually for several hours by heating at 65 °C. The final concentrations of curcumin were in the range of 10^−6^ g/mL to 10^−5^ g/mL. The exact values for all the block copolymers are given in the SIS. The final solutions had a yellow color and were stable for several weeks without any signal of precipitation.

### 2.5. Characterization Techniques

The molecular characteristics [weight average molecular weight (M_w_) and molecular weight distribution, (Ð = M_w_/M_n_)] were measured by size-exclusion chromatography, SEC, using a modular instrument, which consisted of a Waters model 510 pump, U6K sample injector, 401 differential refractometer, and set of 5 μ-Styragel columns with a continuous porosity range from 500 to 10^6^ Å. CHCl_3_ was the carrier solvent at a flow rate of 1 mL/min. Nine polystyrene standards (molecular weights in the range of 970–600,000) were used to calibrate the instrument.

^1^H NMR spectroscopy was employed to calculate the copolymers’ composition. The spectra were recorded in chloroform-d at 30 °C with a 400 MHz Bruker Avance Neo spectrometer (Billerica, MA, USA).

The UV-Vis spectra were recorded using a Perkin Elmer Lamda 650 spectrophotometer operating from 250 to 800 nm, at room temperature, employing a quartz cell of 3 mL.

The T_g_ values of the copolymers were determined by a 2910 Modulated DSC Model from TA Instruments. The samples were heated under a nitrogen atmosphere at a rate of 10 °C/min in the range between −30 °C and 220 °C. The second heating results were obtained in all cases.

Thermogravimetric analysis (TGA) measurements were conducted using a Q50 TGA model from TA Instruments (New Castle, DE, USA). The samples were placed in a platinum pan and heated within the temperature range from room temperature up to 600 °C with a 60 mL/min flow of nitrogen at a heating rate of 10 °C/min.

The measurement of the refractive index increments, *dn*/*dc*, at 25 °C was conducted with a Chromatix KMX-16 (Milton Roy, LDC Division, Riviera Beach, FL, USA) refractometer. The instrument operated at the wavelength of 633 nm and was calibrated with aqueous NaCl solutions.

A Brookhaven Instruments (Holtsville, NY, USA) Bl-200SM Research Goniometer System (Holtsville, NY, USA) was employed to conduct the dynamic light scattering (DLS) measurements. It operated at λ = 640 nm using a laser source at 40 mW of power. The experimentally obtained correlation functions were analyzed both by the cumulant approach and the CONTIN software (Holtsville, NY, USA) [68]. The correlation functions were collected at different angles (45, 90, and 135°), at 25 °C.

The experimentally observed angular dependence of the ratio Γ/q^2^ (Γ being the decay rate of the correlation function and q being the scattering vector) was negligible in the case of the micellar solutions. The apparent translational diffusion coefficients at zero concentration, D_o,app_, were calculated by applying Equation (1):

D_app_ = D_o,app_(1 + kDc) (1)

*k_D_* is the coefficient of the concentration dependence of the diffusion coefficient. The apparent hydrodynamic radii at infinite dilutions, *R_h_*, were calculated using the Stokes–Einstein Equation (2):

R_h_ = kT/6πη_s_D_0,app_ (2)

*k* is Boltzmann’s constant, *T* is the absolute temperature, and *η_s_* is the viscosity of the solvent.

Different protocols were followed for the preparation of the stock solutions for the light scattering measurements. The solutions in CHCl_3_ and THF were prepared by the direct dissolution of the block copolymers in the respective solvent. Thermal treatment at 40 °C overnight was then employed to confirm the formation of stable equilibrium structures. The preparation of the micellar solutions in an aqueous environment involved the formation of solutions in THF. After complete dissolutions, the desired amount of water was gradually added, followed by the heating of the solutions at 50 °C for several hours, thus facilitating complete dissolution. Finally, further heating at 60 °C was employed for the gradual evaporation of THF. For all measurements, the solutions were filtered through 0.22 µm-pore-size filters.

## 3. Results and Discussion

### 3.1. Synthesis of Phthalimidylmethyl Dithiobenzoate, CTA XM

**Scheme 1:**
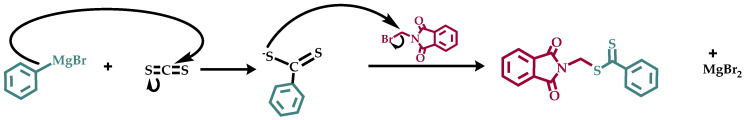
Synthesis of phthalimidylmethyl dithiobenzoate (CTA XM).

It is well known that in RAFT polymerization, monomers are divided into two distinct families, the more activated monomers, MAMs, and the less activated monomers, LAMs, according to their tendency to stabilize the formation of radicals [51,52]. NVP belongs to the category of LAMs, whereas HMA and SMA belong to MAMs. The polymerization of these monomers requires different CTAs with variable ability to stabilize radicals. In fact, CTA1 is a well-known xanthate, which has been frequently employed for the polymerization of LAMs. CTA XM has not been reported so far in the literature. The synthesis was conducted through the reaction given in Scheme 1. The crude product was purified through column chromatography and the yield was about 80%. The synthesis was verified by NMR spectroscopy. The ^1^H NMR spectrum is given in the SIS (Figure S1), confirming the preparation of the target product.

### 3.2. Synthesis of Block Copolymers PNVP-b-PHMA and PNVP-b-PSMA

The RAFT methodology was adopted for the preparation of the PNVP-*b*-PHMA and PNVP-*b*-PSMA block copolymers. The procedure was not a trivial task, since NVP and methacrylates belong to different families of monomers. On the one hand, NVP is considered a LAM, since it is not possible to stabilize radicals in its molecule. On the other hand, methacrylates have the ability to stabilize radicals and therefore belong to the family of MAMs. This differentiation is crucial, since there are specific groups of CTAs that are suitable for each category of monomers. For example, xanthates and dithiocarbamates can be efficiently employed for the polymerization of LAMs, whereas dithioesters and trithiocarbonates are used for the polymerization of MAMs. There are also a family of CTAs that are considered universal, since they are efficient for both MAMs and LAMs, and a family of switchable CTAs that afford similar conclusions after simple chemical transformation (such as protonation under acidic conditions). The combination of both MAMs and LAMs in the same copolymeric structure is not a common procedure and requires special treatment, such as the use of universal or switchable CTAs.

Previously, the preparation of block copolymers between PNVP and either poly(benzyl methacrylate), PBzMA [62], or poly(isobornyl methacrylate), PIBMA [63], has been reported by RAFT copolymerization. In the case of the PIBMA-*b*-PNVP block copolymers, 2-cyanoprop-2-yl-1-dithionaphthalene (CPDN) was applied as CTA, leading to excellent control over the RAFT polymerization of MAMs, such as IBMA. However, in the case of LAMs, it was not effective, leading to poor control. However, the presence of 1,1,1,3,3,3-hexafluoro-2-propanol (HFIP) as the solvent may have been able to promote the efficient polymerization of NVP, finally leading to well-defined block copolymers. The block copolymers with PBzMA were synthesized by the sequential addition of monomers starting from the polymerization of NVP. O-ethyl S-(phthalimidylmethyl) xanthate was employed as the CTA, as a universal CTA. The specific CTA provided excellent control for the RAFT polymerization of NVP and LAMs in general. The subsequent polymerization of BzMA from the originally prepared PNVP macro-CTA afforded the desired block copolymer.

**Scheme 2:**
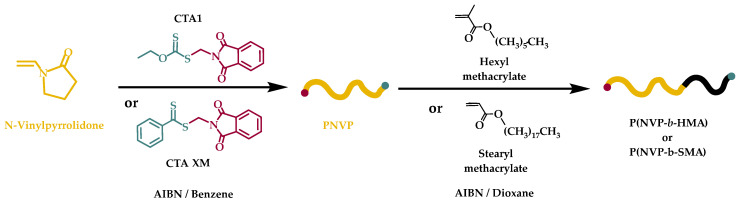
Synthesis of block copolymers PNVP-b-PHMA and PNVP-b-PSMA.

**Figure 1:**
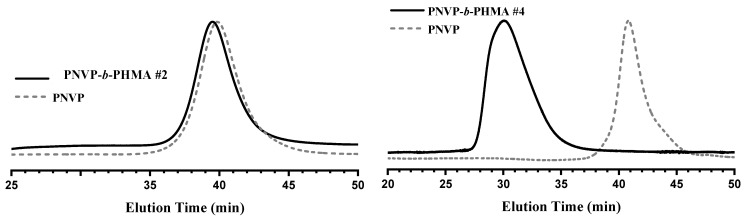
SEC traces from the synthesis of the block copolymers PNVP-*b*-PHMA #2 and #4.

**Figure 2:**
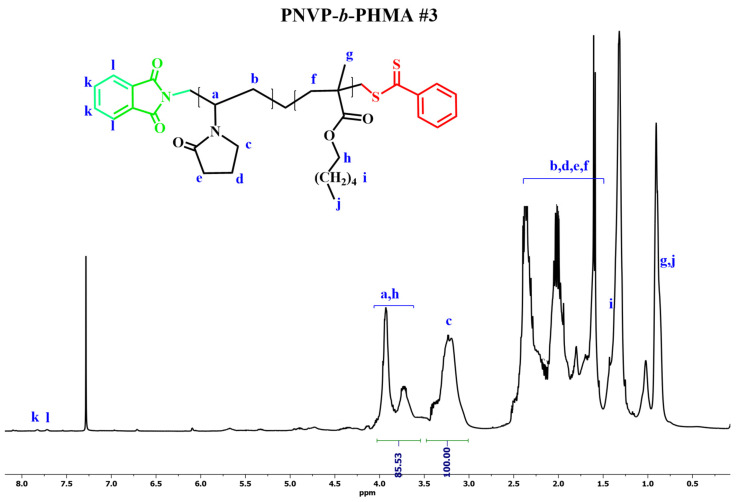
^1^H NMR spectrum for PNVP-*b*-PHMA #3 in CDCl_3_.

**Figure 3:**
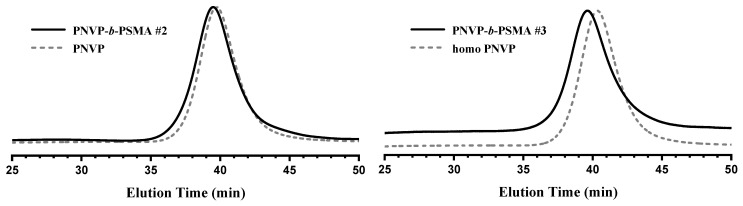
SEC traces from the synthesis of the block copolymers PNVP-*b*-PSMA #2 and #3.

**Figure 4:**
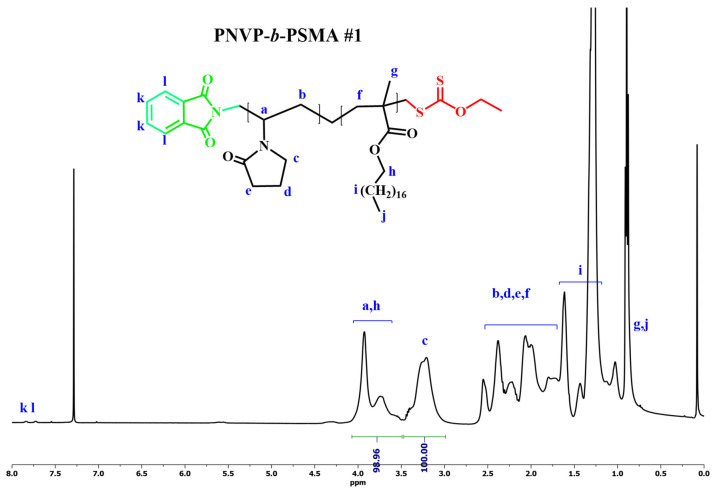
^1^H NMR spectrum for PNVP-*b*-PSMA #1 in CDCl_3_.

This procedure was also employed in the current work. The reaction sequence is given in Scheme 2, whereas the molecular characteristics of the synthesized block copolymers are in Table 1 and Table 2 for the PNVP-*b*-PHMA and PNVP-*b*-PSMA samples, respectively. The reaction sequence was monitored by SEC and ^1^H NMR spectroscopy. Characteristic examples are given in Figure 1, Figure 2, Figure 3 and Figure 4, whereas data for the remaining samples are given in the SIS (Figures S2–S8). The polymerization of NVP was well controlled using CTA1, as was evidenced by the relatively low dispersity values (less than 1.30) and the symmetric peaks from SEC analysis. As was mentioned earlier, CTA1 and, therefore, the corresponding PNVP macro-CTA are not very efficient for the polymerization of methacrylates. This event had two consequences for the obtained copolymers: the first had to do with the SEC traces. They were symmetric, without important tailing effects, but in certain cases, the crude product gave a bimodal distribution, indicating either the presence of termination reactions during the polymerization of the methacrylate monomer or a non-very efficient crossover reaction from the first PNVP block to the final block copolymer.

Therefore, SEC analysis of the block copolymers PNVP-*b*-PHMA #1 and PNVP-*b*-PSMA #1 revealed the presence of excess remaining PNVP block. The samples were purified from the remaining PNVP block in the final product by fractionation using chloroform/methanol as the solvent/non-solvent system. The second consequence of using CTA1 for these experiments was that in order to minimize the broadening of the dispersity of the copolymers, the yield of polymerization was not allowed to reach high levels. It was lower than 60% in all cases.

For the synthesis of the copolymers PNVP-*b*-PHMA #3 and #4, the newly synthesized CTA XM was employed. The specific CTA is, in principle, suitable for the polymerization of MAMs. Consequently, the dispersity values for the PNVP blocks were found to be higher than in the other cases, and the yield was not quantitative. In addition, the SEC traces were not always symmetric, meaning that it was possible to have a substantial amount of PNVP chains without having the desired end-groups coming from the CTA XM reagent. For this reason, the sample PNVP-*b*-PHMA #4 had to be purified by fractionation, as reported earlier. However, the polymerization of the second methacrylate block was well controlled, giving the advantage of synthesizing copolymers with higher methacrylate contents.

The synthesis of the targeted products was confirmed by NMR spectroscopy. The signals of all the protons were traced in the spectra. It was possible to calculate the composition of the copolymers using the signals of the -CH methine proton of the backbone and the -CH_2_ methylene protons of the pyrrolidone ring that are adjacent to the nitrogen atom, along with the -COO-CH_2_ methylene protons of the ester group of the methacrylate monomer.

### 3.3. Thermal Properties

**Figure 5:**
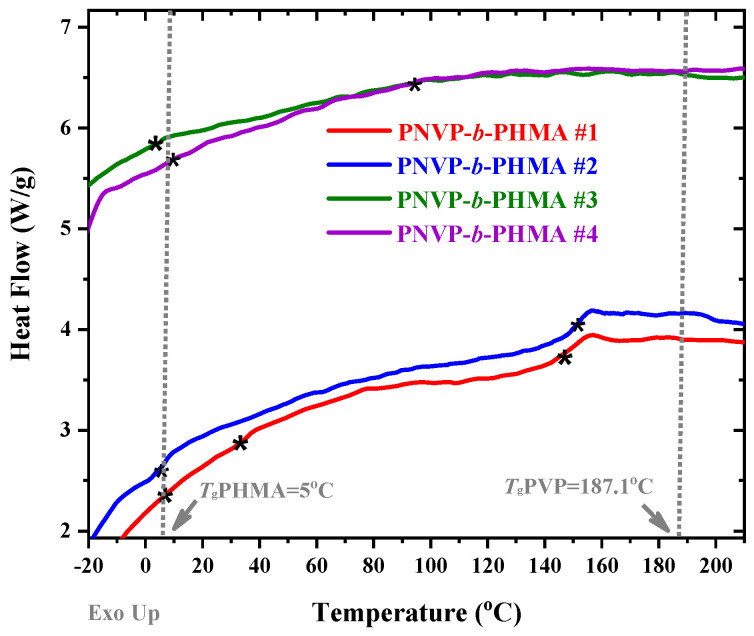
DSC thermograms of the PNVP-b-PHMA block copolymers. * indicates the thermal transition.

**Figure 6:**
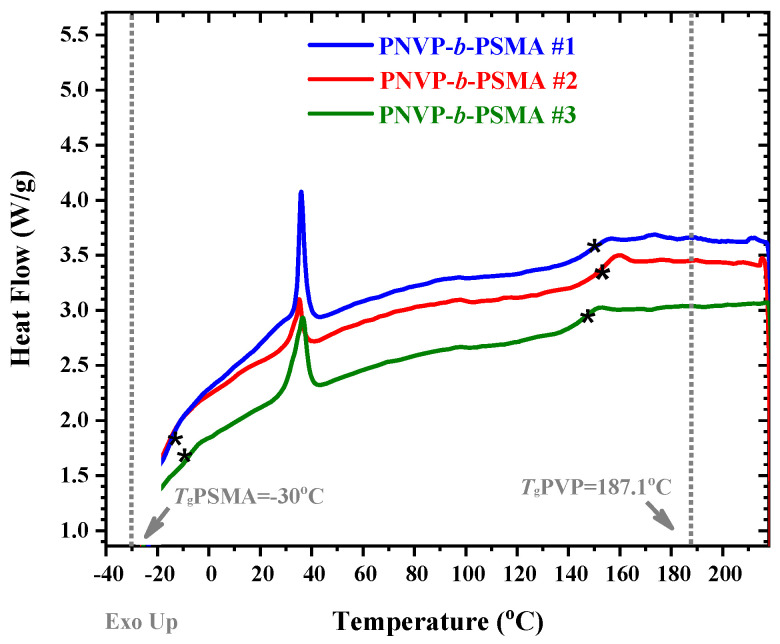
DSC thermograms of the PNVP-b-PSMA block copolymers. * indicates the thermal transition.

The thermal properties of the two families of copolymers were studied by DSC and TGA measurements. The data from the DSC experiments are given in Table 3 and Table 4 and Figure 5 and Figure 6 for the PNVP-b-PHMA and PNVP-b-PSMA block copolymers, respectively.

Both PNVP and PHMA homopolymers were amorphous, with Tg values equal to 187.1 [69] and 5 °C [70,71], respectively. The lower value of PHMA is attributed to the flexible alkyl side chain of the polymethacrylate chains. The block copolymers showed two thermal transitions, indicating that there was microphase separation in the system. However, the obtained Tg values were shifted from the corresponding values of the pure homopolymers, thus revealing the presence of partial mixing in the system. Only sample #1 showed an intermediate weak transition due to the partial mixing of the two phases. This result is attributed to the rather low molecular weights of the respective blocks and the obviously not very high value of the χ parameter of Flory, which did not allow phase separation in low-molecular-weight blocks.

The presence of the large alkyl side chain of the ester group in PSMA led to side-chain crystallization, and therefore, the homopolymer was semi-crystalline, showing a melting temperature of 34 °C and a glass transition temperature of −30 °C [72,73,74]. The PNVP-b-PSMA block copolymers were also microphase-separated, with a first-order transition upon heating, attributed to the melting of the crystalline domains of the polymethacrylate block and two glass transitions from the amorphous regions of the respective blocks. The Tm values were slightly increased compared to in the homopolymer, since the glassy phase of PNVP offered further stabilization to the PSMA crystalline regions. On the contrary, the enthalpy of melting and, consequently, the degree of crystallization were reduced compared to in the homopolymer. This behavior is rather common in amorphous/semi-crystalline block copolymers, indicating that amorphous blocks prevent the organization of semi-crystalline domains [74,75]. The low Tg values were shifted to higher temperatures, whereas the high Tg values became lower, indicating a partial mixing of the two phases, as discussed previously.

**Figure 7:**
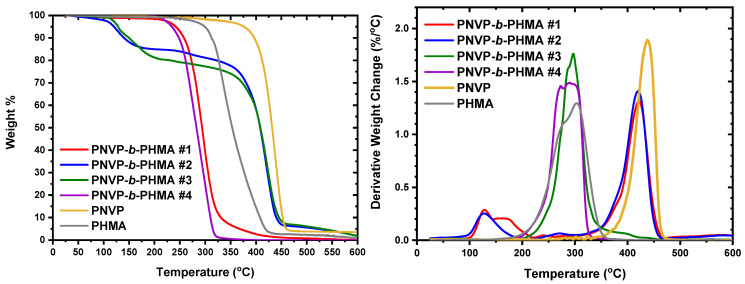
TGA (**left**) and DTG (**right**) thermograms for the PNVP-*b*-PHMA block copolymers and respective homopolymers.

**Figure 8:**
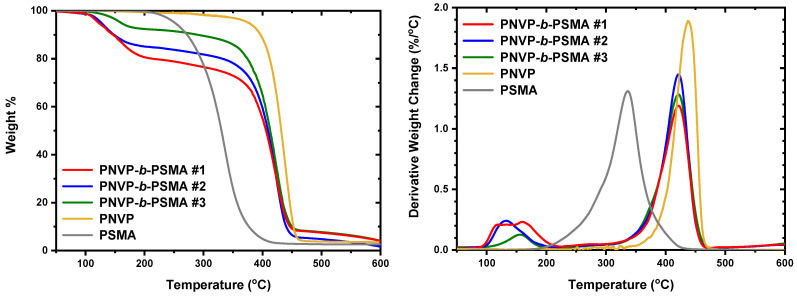
TGA (**left**) and DTG (**right**) thermograms for the PNVP-*b*-PSMA block copolymers and respective homopolymers.

TGA measurements were employed to study the thermal stability of the respective homopolymers and block copolymers. The experimental results are shown in Table 5 and Table 6, whereas the TGA and DTG thermograms for all samples are provided in Figure 7 and Figure 8.

As was found for other polymethacrylates, PNVP was much more thermally stable than PHMA [76]. DTG analysis revealed that a single decomposition peak was observed for the PNVP homopolymer in the range of 348 and 484 °C with a maximum rate of degradation at 438 °C. Similar analysis for PHMA showed that the decomposition pattern was a single peak with a maximum rate at 299 °C, with a shoulder at lower temperatures. The range of decomposition was between 191 and 357 °C. This is direct evidence that the degradation of the side ester group and the main chain of the polymethacrylate took place at similar ranges of temperatures.

A much more complex degradation profile was found for PBzMA [62] and especially for PIBMA [63], where a multistep procedure was traced, indicating that the chemical structure of the ester group of the polymethacrylate block played a crucial role in the determination of the thermal stability of the polymer and the exact mechanism of thermal decomposition. The block copolymers combined the characteristics of the respective homopolymers. The sample PNVP-b-PHMA #4 with the highest composition of PHMA behaved more or less as the respective homopolymer with a single decomposition peak at 292 °C. Sample #3 with 26% moles of HMA had a maximum rate of decomposition at a temperature of 297 °C, which was slightly higher than that of the PHMA homopolymer. However, the range of decomposition was broader compared to that of the homopolymer and sample #4, as a result of the presence of the more thermally stable PNVP block. The block copolymers #1 and #2 with much higher compositions of PNVP had a single degradation peak, which was closer to that of the PNVP block, although relatively lower (421 °C for both blocks compared to 438 °C for the homopolymer).

On the other hand, PSMA was thermally more stable than PHMA, due to the side-chain crystallization of the long alkyl side ester groups. The range of decomposition temperatures was very broad, certainly higher in PSMA rather than in PHMA, showing that the possible mechanism of degradation was similar but the bulky and crystalline-forming steryl group offered more thermal stability to the homopolymer. The block copolymers PNVP-b-PSMA, rich in NVP, had thermal degradation behavior similar to that of PNVP, showing a single decomposition peak with a maximum in the range of 419 to 424 °C. These temperatures were slightly lower than those of the pure PNVP homopolymer, due to the presence of the less thermally stable PSMA block.

For both types of block copolymers, minor peaks at low temperatures, slightly higher than 100 °C, were attributed to humidity in the samples and to remaining traces of organic solvents from the synthetic procedure.

### 3.4. Self-Assembly Behavior of PNVP-b-PHMA Block Copolymers in THF and Aqueous Solutions

**Figure 9:**
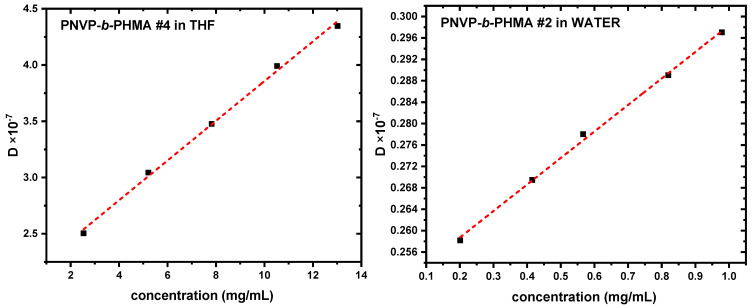
DLS plots of the samples PNVP-*b*-PHMA #4 in THF and #2 in aqueous solution.

The self-assembly behavior of the PNVP-*b*-PHMA block copolymers was studied in CHCl_3_, a common good solvent for both components, in THF, a selective solvent for the PHMA blocks, and in aqueous solutions, since water is a selective solvent for PNVP blocks. Static, SLS, and dynamic light scattering, DLS, techniques were employed to investigate the aggregation of the block copolymers. The SLS data of the PNVP-*b*-PHMA block copolymers in both the common good and the selective solvents are reported in Table 7, whereas the DLS results are in Table 8. Characteristic Zimm plots from the SLS measurements and diffusion coefficient, D, vs. concentration, c, plots from the DLS studies are given in Figure 9 and Figures S9 and S10. In previous studies, regarding the association behavior of block copolymers bearing PNVP blocks, it was shown that THF, although a selective solvent for PNVP, is not able to promote strong aggregation effects, leading to low degrees of association, N_w_. Especially for the PNVP-*b*-PBzMA block copolymers, the almost exclusive formation of unimolecular micelles was concluded [62]. In the case of the PNVP-*b*-PIBMA blocks, slightly higher N_w_ values were obtained [63]. Similar results were obtained in the present study with the PNVP-*b*-PHMA block copolymers in THF. The aggregation numbers were very low (N_w_ < 10), indicating the formation of unimolecular micelles or small spherical micelles. It is important to mention that the copolymers with the highest PNVP content (PNVP-*b*-PHMA samples #1, #2, and #3) had slightly lower N_w_ values than sample #4, which contained only 5% mol of PNVP. In this case, compact star-like micelles prevailed in THF solutions. The low second virial coefficient values, A_2_, in THF solutions confirmed the formation of unimolecular or low-association-number micelles. Therefore, it can be concluded that the ability of THF to form large associates in these PNVP-based block copolymers was not very pronounced.

These results were further confirmed by the DLS measurements. CONTIN analysis revealed the presence of single populations in THF solutions. The absence of angular dependence verified the formation of spherical structures, which were relatively polydisperse, since the polydispersity factor μ_2_/Γ^2^ (μ_2_ being the second moment of the cumulant analysis and Γ being the decay rate of the correlation function) was higher than 0.1 for all samples. These values were comparable for both the CHCl_3_ and the THF solutions. Characteristic plots are given in the SIS (Figures S11 and S12). The R_h_ values in THF were lower than those measured in CHCl_3_ for all samples, supporting the conclusion derived from the SLS measurements regarding the formation of unimolecular or small and compact aggregates in THF. These findings were further confirmed by the D vs. c plots, which were linear with small k_D_ values. In addition, the k_D_ values in THF were lower than those in CHCl_3_. This result is reasonable, due to the relationship between k_D_ and A_2_ through the following equation:

k_D_ = 2A_2_M + k_f_ − u (3)

M is the molecular weight, k_f_ is the coefficient of the concentration dependence of the friction coefficient, and u is the partial specific volume of the polymer. The low values of A_2_ in THF implied low k_D_ values as well.

Completely different behavior was obtained in aqueous solutions. The data from SLS and DLS measurements are given in Table 7 and Table 8, respectively. The sample PNVP-*b*-PHMA #4, due to the very low PNVP content, was not soluble in water despite the various protocols employed for the preparation of the solutions. In an effort to obtain equilibrium micellar structures, the copolymers were initially dissolved in THF, where, as previously discussed, unimolecular micelles or supramolecular structures with very low aggregation numbers exist. Water was then gradually added, followed by the heating of the mixture at 50 °C, leading to the re-organization of the supramolecular entities. Subsequent further heating at 60 °C allowed the gradual evaporation of the volatile THF and thus led to the formation of equilibrium micellar structures in the aqueous environment.

Very high aggregation numbers were measured by SLS measurements, despite the fact that the content of the copolymers in the soluble PNVP was very high. Judging from these strong association phenomena, it was reasonable to expect the very low A_2_ values that were measured. These values were an order of magnitude lower than those measured in THF, where association was also effective. In addition, very large radii of gyration, Rg, values were measured in aqueous solutions, much higher than those in THF solutions. A comparison of these Rg values with the huge aggregation numbers leads to the conclusion that very compact supramolecular structures exist in aqueous solutions.

Further elucidation of this situation was offered by DLS measurements. Characteristic D vs. c plots are given in Figure 9. CONTIN analysis revealed the presence of single populations of associates in all concentrations and for all the samples with much higher R_ho_ values compared to those measured in CHCl_3_ and THF. Representative CONTIN plots are given in Figure S13. These populations had lower polydispersity factor (μ_2_/Γ^2^ < 0.2) values compared to the corresponding values in the common good solvent, CHCl_3_, and in THF. Furthermore, no angular dependence was found, and the populations were thermally stable up to 60 °C without any signal of disassociation or the formation of higher-dimension supramolecular structures. Finally, the R_g_/R_h_ ratios coming from SLS and DLS measurements were close to unity for all samples examined in aqueous solutions. All these findings lead to the same conclusion that thermally stable, spherical, and compact micelles are present in water. The k_D_ values were much higher in water than in THF for the aggregating systems, due to the extremely high molecular weight of the micellar entities.

A comparison of the micellization behavior of the present system with the corresponding PNVP-*b*-PBzMA [62] and PNVP-*b*-PIBMA block copolymer micelles [63] in THF and water revealed the presence of both similarities and differences. In THF, a solvent selective for polymethacrylate blocks, the behavior was more or less the same, indicating that the core-forming PNVP block was responsible for the experimental results and that the exact chemical nature of the corona-forming polymethacrylate block did not play a crucial role. Unimolecular or small spherical micelles with very low aggregation numbers were formed in THF. The situation was different in aqueous solutions. Polymethacrylates were the core-forming blocks, and therefore, the nature of the side ester group had a significant impact on the association behavior. In the case of the PNVP-*b*-PIBMA block copolymers, equilibrium between micelles and clusters was established. On the contrary, compact, spherical micelles with relatively low polydispersity values were formed in the other cases. The aggregation numbers of the micelles formed from the PNVP-*b*-PBzMA block copolymers were lower than those measured for the respective PNVP-*b*-PHMA block copolymers, despite the fact that both the molecular weight and the content of the PBzMA component were, in most samples, much higher than those of the corresponding PHMA. This indicates that the more flexible linear n-hexyl group of PHMA facilitated the accommodation of the polymethacrylate chains to larger and compact cores, leading to the formation of micelles with very high N_w_ values.

### 3.5. Self-Assembly Behavior of PNVP-b-PSMA Block Copolymers in THF and Aqueous Solutions

**Figure 10:**
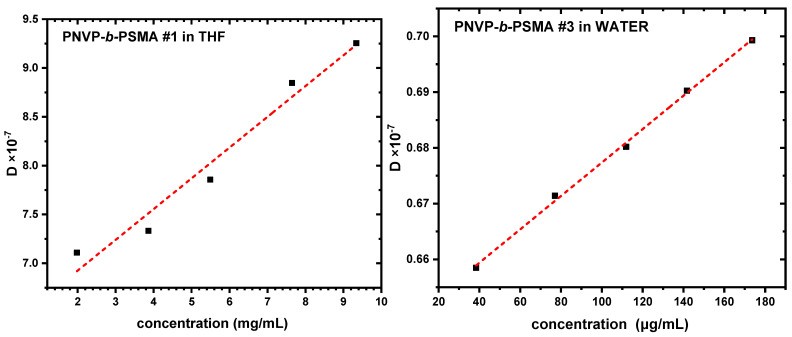
DLS plots of the samples PNVP-*b*-PSMA #1 in THF and #3 in aqueous solution.

The micellization properties of the PNVP-*b*-PSMA block copolymers were studied in THF and aqueous solutions by employing SLS and DLS measurements. The experimental results are provided in Table 9 and Table 10, whereas representative plots are in Figure 10. The general picture was similar to that mentioned for the PNVP-*b*-PHMA block copolymers, since the chemical nature of both the hydrophilic and the hydrophobic compounds were almost identical. The only difference was due to the size of the ester group of the polymethacrylate block. The stearyl group of PSMA was more hydrophobic than the hexyl group of PHMA. This event was expected to further promote the association of the PNVP-*b*-PSMA block copolymers in aqueous solutions. However, the stearyl group was bulkier than the hexyl group, thus preventing the organization of extended cores and leading to lower aggregation numbers. The experimental findings from SLS measurements revealed that the steric hindrance effects prevailed and the N_w_ values for the PSMA-containing copolymers, although large enough, were lower than those obtained for both PNVP-*b*-PHMA and PNVP-*b*-PBzMA block copolymers. Therefore, the manipulation of the chemical structure of the ester group of the polymethacrylate chain may have altered the self-assembly behavior.

In THF, only unimolecular or small and compact aggregates were formed, with R_h_ values lower than those measured in the common good solvent, CHCl_3_. On the other hand, spherical, compact, and large micellar structures were obtained in aqueous solutions. CONTIN analysis from the DLS experiments showed that single supramolecular populations existed in the water with relatively low polydispersity factor values (μ_2_/Γ^2^ < 0.2) for all samples and all concentrations examined (Figure S14). The ratio R_g_/R_ho_ was close to unity in aqueous solutions.

### 3.6. Encapsulation of Curcumin into Micellar Solutions

**Scheme 3:**
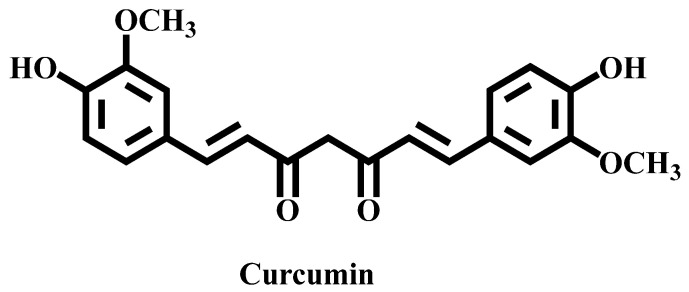
Structure of curcumin.

One of the most important applications of the self-assembled structures formed by amphiphilic block copolymers in aqueous solutions is the encapsulation of hydrophobic compounds within the micellar core. Emphasis is given on the encapsulation of compounds with biological and pharmaceutical activity for gene or drug delivery studies. Amphiphilic blocks bearing PNVP as the water-soluble constituent have been reported in several works. In previous studies the encapsulation of curcumin was examined in the block copolymers PNVP-*b*-PBzMA and PNVP-*b*-PIBMA. Curcumin (Scheme 3) is an interesting compound suitable for these studies for two main reasons. The first one is that it exhibits various interesting pharmaceutical activities, with antioxidant, antibacterial, antimicrobial, antifungal, anti-inflammatory, and anti-carcinogenic properties [77,78,79,80]. Therefore, it has been applied for the treatment of several diseases, such as Alzheimer’s disease, multiple myeloma, psoriasis, myelodysplastic syndrome, and anti-human immunodeficiency virus cycle replication. The second reason is that the encapsulation of curcumin can be easily studied by UV-Vis spectroscopy, since the solutions of curcumin have a characteristic yellow–orange color. Subsequently, it is quite understandable why multiple efforts have been devoted to achieving the efficient encapsulation of curcumin in block copolymer micelles [81].

Curcumin has a characteristic absorbance band at 423.50 nm in THF solutions. A calibration curve from the absorbance values with concentration was recorded and the results are provided in the SIS (Figure S15). Employing this calibration curve and measuring the absorbance of the polymer solutions with the encapsulated curcumin, the drug loading capacity, DLC, and the drug loading efficiency, DLE, were calculated by employing the following Equations (3) and (4):

(4) $$drug\;loading\;capacity\;\left(DLC\right)\%=\frac{mass\;of\;loaded\;drug}{mass\;of\;polymer}\times 100$$

(5) $$drug\;loading\;efficiency\;\left(DLE\right)\%=\frac{mass\;of\;loaded\;drug}{mass\;of\;drug\;in\;feed}\times 100$$

**Figure 11:**
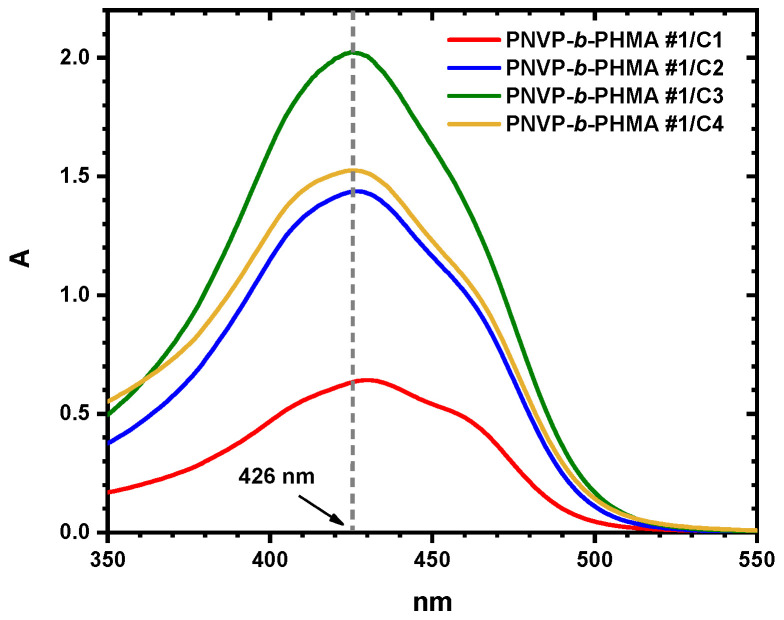
UV-Vis spectra of the sample PNVP-*b*-PHMA #1 with varying concentrations of curcumin (polymer concentration ~5.2 × 10^−4^ g/mL and curcumin concentration from 3 × 10^−6^ up to 1.0 × 10^−5^ g/mL).

**Figure 12:**
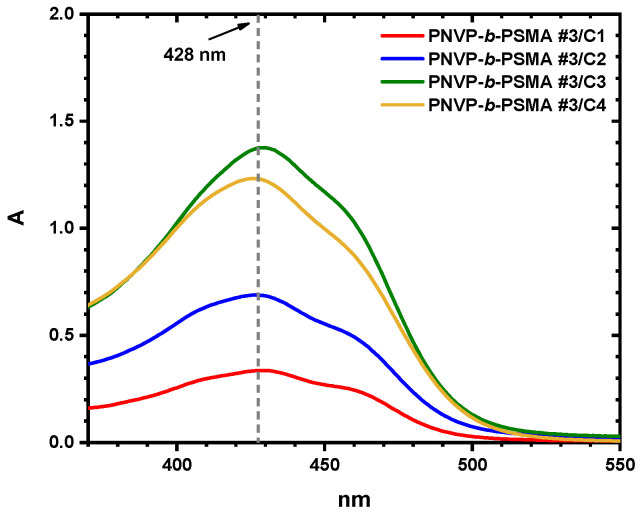
UV-Vis spectra of the sample PNVP-*b*-PSMA #3 with varying concentrations of curcumin (polymer concentration ~5.3 × 10^−4^ g/mL and curcumin concentration from 1.4 × 10^−6^ up to 6.8 × 10^−6^ g/mL).

Characteristic UV-Vis spectra from the encapsulation of various amounts of curcumin within the micellar core of the block copolymers PNVP-*b*-PHMA #1 and PNVP-*b*-PSMA #3 are given in Figure 11 and Figure 12, whereas the complete data with the DLC and DLE values are given in Table 11 and Table 12. Additional data are given in the SIS (Figures S16 and S17).

It is evident that the encapsulation of curcumin was efficient in the PNVP-*b*-PHMA solutions. The sample PNVP-*b*-PHMA #2 had lower DLC and DLE values compared to the other samples, since it had the lowest molecular weight and, therefore, the micelles did not have very extended cores for the encapsulation of larger quantities of curcumin. In general, the DLC values for the same concentration of the block copolymer progressively increased upon increasing the concentration of curcumin, whereas the DLE values initially increased and gradually reached a plateaued value. These values were considerably lower than those found for the PNVP-*b*-PBzMA copolymers, indicating that the steric hindrance of the ester group of the hydrophobic polymethacrylate block greatly affected the entrapment ability for curcumin. This was further manifested in the case of the PNVP-*b*-PSMA block copolymers, where the steric hindrance was even more pronounced, and the micellar cores were more compact, leading to even lower DLC and DLE values.

## 4. Conclusions

Block copolymers of N-vinyl pyrrolidone (NVP) with n-hexyl methacrylate (HMA, PNVP-*b*-PHMA) and stearyl methacrylate (SMA, PNVP-*b*-PSMA) were prepared by the RAFT methodology and sequential addition of monomers, starting from the polymerization of NVP and employing either O–ethyl S–(phthalimidylmethyl) xanthate, CTA1, or phthalimidylmethyl dithiobenzoate, CTA XM, as universal CTAs. CTA XM was prepared for the first time and offered the possibility to synthesize block copolymers with a high composition of PNVP. In the first case, block copolymers with two amorphous blocks with low and high Tg values were obtained. In the other case, blocks with an amorphous and a semi-crystalline component were obtained. Relatively well-defined products were obtained. The study of the thermal properties of the copolymers revealed that the copolymers were microphase-separated. However, partial mixing was observed. The thermal stability of the block copolymers was determined by both components. The micellization behavior of the block copolymers was studied in THF, which was a selective solvent for the polymethacrylate blocks, and in aqueous solutions, where PNVP was soluble, employing static, SLS, and dynamic light scattering, DLS, techniques. In THF, unimolecular micelles or supramolecular structures with a low degree of aggregation were observed for both types of block copolymers. On the other hand, stable, compact, and spherical micelles with very high degrees of association were obtained in aqueous solutions. The bulky nature of the stearyl side ester group introduced severe steric hindrance effects, leading to lower association numbers in the case of the PNVP-*b*-PSMA block copolymers compared to the corresponding PNVP-*b*-PHMA. Consequently, the manipulation of the nature of the methacrylate’s ester group may have considerably altered the self-assembly behavior. The efficient encapsulation of curcumin within the micellar core of the supramolecular structures was demonstrated by UV-Vis spectroscopy measurements. The PNVP-*b*-PSMA block copolymers showed a smaller ability to encapsulate curcumin, due to their lower degrees of association and their more compact micellar cores. Therefore, it seems possible to gradually replace poly(ethylene oxide) as the water-soluble block in amphiphilic copolymers, thus avoiding side effects that have been verified in the literature.

## Figures and Tables

**Table 1:** Molecular characteristics of block copolymers PNVP-b-PHMA.

	Macro-CTA (PNVP) *		Block Copolymers *		NVP	HMA
Sample	M_n_ 10^3^ (Daltons)	Ð	M_n_ 10^3^ (Daltons)	Ð	% mol **	% mol **
PNVP-*b*-PHMA #1	29.8	1.29	36	1.26	87	13
PNVP-*b*-PHMA #2	10.6	1.26	13	1.27	84	16
PNVP-*b*-PHMA #3	27.0	1.60	32	1.68	74	26
PNVP-*b*-PHMA #4	6.3	1.34	130	1.53	5	95

* By SEC in CHCl_3_. ** By ^1^H-NMR.

**Table 2:** Molecular characteristics of block copolymers PNVP-b-PSMA.

	Macro-CTA (PNVP) *		Block Copolymers *		NVP	SMA
Sample	M_n_ 10^3^ (Daltons)	Ð	M_n_ 10^3^ (Daltons)	Ð	% mol **	% mol **
PNVP-*b*-PSMA #1	29.8	1.29	37	1.31	67	33
PNVP-*b*-PSMA #2	7.9	1.28	9.3	1.35	94	6
PNVP-*b*-PSMA #3	7.9	1.28	10.8	1.31	83	17

* By SEC in CHCl_3_. ** By ^1^H-NMR.

**Table 3:** DSC results of the PNVP-*b*-PHMA block copolymers.

Sample	*T_g_* Experimental (°C)		
**PNVP-*b*-PHMA #1**	11.4	36.7	151.3
**PNVP-*b*-PHMA #2**	7.1	-	152.8
**PNVP-*b*-PHMA #3**	5.5	-	118.1
**PNVP-*b*-PHMA #4**	18.5	-	114.6
**PNVP**	-	-	187.1
**PHMA**	5	-	-

**Table 4:** DSC results of the PNVP-*b*-PSMA block copolymers.

Sample	W_SMA_ %	*T*_m_ (°C)	ΔH(j/g _block_)	ΔH (j/g_SMA_)	X_c_ %	*T*_g1_ (°C)	*T*_g2_ (°C)
**PNVP-*b*-PSMA #1**	59.9	35.9	31.6	52.7	69.4	−14.8	147.0
**PNVP-*b*-PSMA #2**	16.2	35.2	9.6	59.2	77.9	−15.9	154.7
**PNVP-*b*-PSMA #3**	38.4	36.6	22.3	58.0	76.4	−7.9	147.2
**PNVP**	-	-	-	-	-	-	187.1
**PSMA**	-	34.0	-	75.9	-	−30	-

**Table 5:** TGA results of the PNVP-*b*-PHMA block copolymers.

Sample	Start1	End1	Max1 (°C)	Start2	End2	Max2 (°C)
**PNVP-*b*-PHMA #1**	94.9	220.4	128.7 (broad)	320.2	466.1	420.9
**PNVP-*b*-PHMA #2**	94.9	194.5	127.7	318.9	463.8	421.0
**PNVP-*b*-PHMA #3**	204.3	413.2	296.9	-	-	-
**PNVP-*b*-PHMA #4**	188.3	333.0	291.5 (broad)	-	-	-
**PNVP**	-	-	-	347.63	484.06	437.53
**PHMA**	190.8	356.6	299.1 (shoulder)	-	-	-

**Table 6:** TGA results of the PNVP-*b*-PSMA block copolymers.

Sample	Start1	End1	Max1 (°C)	Start2	End2	Max2 (°C)
**PNVP-*b*-PSMA #1**	88.8	216.6	140.8 (broad)	339.4	463.7	419.3
**PNVP-*b*-PSMA #2**	94.3	209.2	129.1	312.6	469.2	423.5
**PNVP-*b*-PSMA #3**	113.4	198.9	154.8	323.6	470.8	421.0
**PNVP**	-	-	-	347.6	484.0	437.5
**PSMA**	185.9	430.3	335.8	-	-	-

**Table 7:** SLS data for the PNVP-*b*-PHMA block copolymers.

Sample	Solvent	M_W_ From SEC	M_w_ From SLS	N_w_	R_g_ (nm)	A (cm^3^mol/g^2^)
**PNVP-*b*-PHMA #1**	CHCl_3_	36 × 10^3^				
**PNVP-*b*-PHMA #1**	THF		5.59 × 10^4^	1.55		2.20 × 10^−4^
**PNVP-*b*-PHMA #1**	WATER		5.88 × 10^7^	1633	59.9	2.96 × 10^−5^
**PNVP-*b*-PHMA #2**	CHCl_3_	13 × 10^3^				
**PNVP-*b*-PHMA #2**	THF		2.84 × 10^4^	2.18		1.75 × 10^−4^
**PNVP-*b*-PHMA #2**	WATER		3.58 × 10^8^	27,538	103.6	9.98 × 10^−6^
**PNVP-*b*-PHMA #3**	CHCl_3_	32 × 10^3^				
**PNVP-*b*-PHMA #3**	THF		1.32 × 10^5^	4.13		4.20 × 10^−5^
**PNVP-*b*-PHMA #3**	WATER		8.06 × 10^8^	25,187	124.0	1.18 × 10^−5^
**PNVP-*b*-PHMA #4**	CHCl_3_	130 × 10^3^				
**PNVP-*b*-PHMA #4**	THF		8.76 × 10^5^	6.74	65.0	1.01 × 10^−3^

**Table 8:** DLS data for the PNVP-*b*-PHMA block copolymers.

Sample	Solvent	Do	K_d_	R_g_ (nm)	R_ho_ (nm)	R_g_/R_ho_
**PNVP-*b*-PHMA #1**	CHCl_3_	6.294 × 10^−7^	23.36		6.41	
**PNVP-*b*-PHMA #1**	THF	7.635 × 10^−7^	19.04		6.20	
**PNVP-*b*-PHMA #1**	WATER	3.469 × 10^−8^	384	59.9	70.73	0.85
**PNVP-*b*-PHMA #2**	CHCl_3_	3.500 × 10^−7^	143		11.53	
**PNVP-*b*-PHMA #2**	THF	9.611 × 10^−7^	37.59		4.93	
**PNVP-*b*-PHMA #2**	WATER	2.488 × 10^−8^	199	103.6	98.62	1.05
**PNVP-*b*-PHMA #3**	CHCl_3_	2.532 × 10^−7^	160		15.94	
**PNVP-*b*-PHMA #3**	THF	5.106 × 10^−7^	33.23		9.28	
**PNVP-*b*-PHMA #3**	WATER	2.106 × 10^−8^	1023	124	116.51	1.06
**PNVP-*b*-PHMA #4**	CHCl_3_	1.621 × 10^−7^	103		24.90	
**PNVP-*b*-PHMA #4**	THF	2.092 × 10^−7^	84.35	65	22.64	2.87

**Table 9:** SLS results of the PNVP-*b*-PSMA block copolymers in CHCl_3_, in THF, and in aqueous solution.

Sample	Solvent	M_w_ From SEC	M_w_ From SLS	N_w_	R_g_ (nm)	A_2_ (cm^3^mol/g^2^)
**PNVP-*b*-PSMA #1**	CHCl_3_	37 × 10^3^				
**PNVP-*b*-PSMA #1**	THF		2.53 × 10^5^	6.84		6.00 × 10^−4^
**PNVP-*b*-PSMA #1**	WATER		7.53 × 10^7^	2035	48.0	4.53 × 10^−5^
**PNVP-*b*-PSMA #2**	CHCl_3_	9.3 × 10^3^				
**PNVP-*b*-PSMA #2**	THF		4.70 × 10^4^	5.05		3.50 × 10^−4^
**PNVP-*b*-PSMA #2**	WATER		3.68 × 10^7^	3957	43.8	7.40 × 10^−5^
**PNVP-*b*-PSMA #3**	CHCl_3_	10.8 × 10^3^				
**PNVP-*b*-PSMA #3**	THF		7.20 × 10^4^	6.67		2.50 × 10^−4^
**PNVP-*b*-PSMA #3**	WATER		1.192 × 10^7^	1104	36.1	7.30 × 10^−5^

**Table 10:** DLS results of the PNVP-*b*-PSMA block copolymers in CHCl_3_, in THF, and in aqueous solution.

Sample	Solvent	Do	K_d_	R_g_ (nm)	R_ho_ (nm)	R_g_/R_ho_
**PNVP-*b*-PSMA #1**	CHCl_3_	3.289 × 10^−7^	158.8		12.27	
**PNVP-*b*-PSMA #1**	THF	6.295 × 10^−7^	50.05		7.53	
**PNVP-*b*-PSMA #1**	WATER	3.171 × 10^−8^	1179	48	77.38	0.62
**PNVP-*b*-PSMA #2**	CHCl_3_	3.116 × 10^−7^	231.2		12.96	
**PNVP-*b*-PSMA #2**	THF	9.033 × 10^−7^	28.90		5.24	
**PNVP-*b*-PSMA #2**	WATER	4.418 × 10^−8^	952.0	43.8	55.53	0.79
**PNVP-*b*-PSMA #3**	CHCl_3_	2.567 × 10^−7^	166.0		15.72	
**PNVP-*b*-PSMA #3**	THF	6.577 × 10^−7^	156.8		7.20	
**PNVP-*b*-PSMA #3**	WATER	6.474 × 10^−8^	462.9	36.1	37.90	0.95

**Table 11:** DLC and DLE values from the encapsulation of curcumin within the micellar solutions of the PNVP-*b*-PHMA #1 block copolymers (polymer concentration ~5.2 × 10^−4^ g/mL and curcumin concentration from 3 × 10^−6^ up to 1.0 × 10^−5^ g/mL).

Sample	DLC%	DLE%
**PNVP-*b*-PHMA #1**		
**1/c1**	0.59	50.54
**1/c2**	1.35	76.50
**1/c3**	1.92	67.96
**1/c4**	1.46	38.58
**PNVP-*b*-PHMA #2**		
**2/c1**	0.12	13.30
**2/c2**	0.78	35.75
**2/c3**	0.82	29.09
**2/c4**	1.01	29.56
**PNVP-*b*-PHMA #3**		
**3/c1**	0.32	34.65
**3/c2**	0.99	48.46
**3/c3**	1.28	47.33
**3/c4**	1.60	48.46

**Table 12:** DLC and DLE values from the encapsulation of curcumin within the micellar solutions of the PNVP-*b*-PSMA #3 block copolymers (polymer concentration ~5.3 × 10^−4^ g/mL and curcumin concentration from 1.4 × 10^−6^ up to 6.8 × 10^−6^ g/mL).

Sample	DLC%	DLE%
**PNVP-*b*-PSMA #1**	-	-
**1/c1**	0.40	39.97
**1/c2**	0.91	40.99
**1/c3**	1.32	46.11
**1/c4**	1.68	47.01
**PNVP-*b*-PSMA #2**		
**2/c1**	0.14	11.88
**2/c2**	0.92	39.21
**2/c3**	0.81	26.74
**2/c4**	1.43	39.57
**PNVP-*b*-PSMA #3**		
**3/c1**	0.27	24.01
**3/c2**	0.61	26.39
**3/c3**	1.26	43.19

## Data Availability

The original contributions presented in this study are included in the article/Supplementary Materials. Further inquiries can be directed to the corresponding author(s).

## Supplementary Materials

The following supporting information can be downloaded at https://www.mdpi.com/article/10.3390/polym17081122/s1. Figure S1. ^1^H NMR spectrum of CTA MX in CHCl_3_; Figure S2. SEC traces of PNVP-b-PHMA #1 and #3; Figure S3. ^1^H NMR spectrum of PNVP-b-PHMA #1 in CDCl_3_; Figure S4. ^1^H NMR spectrum of PNVP-b-PHMA #2 in CDCl_3_; Figure S5. ^1^H NMR spectrum of PNVP-b-PHMA #4 in CDCl_3_; Figure S6. SEC traces of PNVP-b-PSMA #1; Figure S7. ^1^H NMR spectrum of PNVP-b-PSMA #2 in CDCl_3;_ Figure S8. ^1^H NMR spectrum of PNVP-b-PSMA #3 in CDCl_3_; Figure S9. SLS Zimm plot of sample PNVP-b-PHMA #4 in THF; Figure S10. SLS Zimm plot of sample PNVP-b-PHMA #2 in aqueous solution; Figure S11. CONTIN plots of PNVP-b-PHMA #1 (c = 1.400 × 10^−2^ mg/mL) and #2 (c = 1.983 × 10^−2^ mg/mL) in CHCl_3_; Figure S12. CONTIN plots of PNVP-b-PHMA #1 (c = 1.330 × 10^−2^ mg/mL) and #2 (c = 5.665 × 10^−3^ mg/mL) in THF; Figure S13. CONTIN plots of PNVP-b-PHMA #2 (c3 = 5.665 × 10^−4^ mg/mL and c4 = 8.196 × 10^−4^ mg/mL) in water; Figure S14. CONTIN plots of PNVP-b-PSMA #2 (c5 = 1.288 × 10^−4^ mg/mL and c6 = 1.492 × 10^−4^ mg/mL) in water; Figure S15. Curcumin calibration curve; Figure S16. UV-Vis spectra for PNVP-b-PHMA #2 and #3 with encapsulated curcumin; Figure S17. UV-Vis spectra for PNVP-b-PSMA #1 and #2 with encapsulated curcumin. Table S1. Quantities for the synthesis of the PNVP-b-PHMA block copolymers; Table S2. Quantities for the synthesis of the PNVP-b-PSMA block copolymers; Table S3. UV-Vis analysis of PNVP-b-PHMA solutions with encapsulated curcumin; Table S4. UV-Vis analysis of PNVP-b-PSMA solutions with encapsulated curcumin.

## References

[1] Teodorescu M., Bercea M. (2015). Poly(vinylpyrrolidone)—A Versatile Polymer for Biomedical and Beyond Medical Applications. Polym.-Plast. Technol. Eng..

[2] Franco F., De Marco I. (2020). The Use of Poly(N-vinyl pyrrolidone) in the Delivery of Drugs: A Review. Polymers.

[3] Moulay S. (2013). Molecular iodine/polymer complexes. J. Polym. Eng..

[4] Husain M.S.B., Gupta A., Alashwal B.Y., Sharma S. (2018). Synthesis of PVA/PVP based hydrogel for biomedical applications: A review. Energy Sources Part. A Recovery Util. Environ. Eff..

[5] Reis C.P., Silva C., Martinho N., Rosado C. (2013). Drug carriers for oral delivery of peptides and proteins: Accomplishments and future Perspectives. Therap. Deliv..

[6] Hamidi M., Azadi A., Rafiei P. (2008). Hydrogel nanoparticles in drug delivery. Adv. Drug Deliv. Rev..

[7] Dolman M.E.M., Harmsen S., Storm G., Hennink W.E., Kok R.J. (2010). Drug targeting to the kidney: Advances in the active targeting of therapeutics to proximal tubular cells. Adv. Drug Deliv. Rev..

[8] Kurakula M., Koteswara Rao G.S.N. (2020). Pharmaceutical assessment of polyvinylpyrrolidone (PVP): As excipient from conventional to controlled delivery systems with a spotlight on COVID-19 inhibition. J. Drug Deliv. Sci. Technol..

[9] Liu X., Xu Y., Wu Z., Chen H. (2013). Poly(N-vinylpyrrolidone)-Modified Surfaces for Biomedical Applications. Macromol. Biosci..

[10] Haaf F., Sanner A., Straub F. (1985). Polymers of N-Vinylpyrrolidone: Synthesis, Characterization and Uses. Polym. J..

[11] Halake K., Birajdar M., Kim B.S., Bae H., Lee C.-C., Kim Y.J., Kim S., Kim H.J., Ahn S., An S.Y. (2014). Recent application developments of water-soluble synthetic polymers. J. Industr. Engin. Chem..

[12] Koczkur K.M., Mourdikoudis S., Polavarapu L., Skrabalak S.E. (2015). Polyvinylpyrrolidone (PVP) in nanoparticle synthesis. Dalton Trans..

[13] Wiley B., Sun Y., Mayers B., Xia Y. (2005). Shape-Controlled Synthesis of Metal Nanostructures: The Case of Silver. Chem. Eur. J..

[14] Qiu L.Y., Bae Y.H. (2006). Polymer Architecture and Drug Delivery. Pharm. Res..

[15] Karanikolopoulos N., Pitsikalis M., Hadjichristidis N., Georgikopoulou K., Calogeropoulou T., Dunlap J.R. (2007). pH-Responsive aggregates from double hydrophilic block copolymers carrying zwitterionic groups. Encapsulation of antiparasitic compounds for the treatment of leishmaniasis. Langmuir.

[16] Kwon G.S., Okano T. (1996). Polymeric micelles as new drug carriers. Adv. Drug Deliv. Rev..

[17] Karanikolopoulos N., Zamurovic M., Pitsikalis M., Hadjichristidis N. (2010). Poly(DL-lactide)-*b*-Poly(N,N-dimethylamino-2-ethyl methacrylate): Synthesis, characterization, micellization behaviour in aqueous solutions and encapsulation of the hydrophobic drug dipyridamole. Biomacromolecules.

[18] Trubetskoy V.S. (1999). Polymeric micelles as carriers of diagnostic agents. Adv. Drug Deliv. Rev..

[19] Kataoka K., Harada A., Nagasaki Y. (2001). Block copolymer micelles for drug delivery: Design, characterization and biological significance. Adv. Drug Deliv. Rev..

[20] Kakizawa Y., Kataoka K. (2002). Block copolymer micelles for delivery of gene and related compounds. Adv. Drug Deliv. Rev..

[21] Sidorov S.N., Bronstein L.M., Valetsky P.M., Hartmann J., Coelfen H., Schnablegger H., Antonietti M. (1999). Stabilization of metal nanoparticles in aqueous medium by poly(ethylene oxide)-poly(ethylene imine) block copolymers. J. Colloid. Interface Sci..

[22] Cao T., Yin W., Armstrong J.L., Webber S.E. (1994). Adsorption of photoactive amphiphilic polymers onto hydrophobic polymer films polystyrene-b-poly(2-vinylnaphthalene)-b-poly(methacrylic acid). Langmuir.

[23] Spatz J.P., Sheiko S., Möller M. (1996). Ion stabilized block copolymer micelles film formation and inter micellar interaction. Macromolecules.

[24] Lazzari M., Scalarone D., Hoppe C.E., Vazquez-Vazquez C., Lòpez-Quintela M.A. (2007). Tunable polyacrylonitrile based micellar aggregates as a potential tool for the fabrication of carbon nanofibers. Chem. Mater..

[25] Dau H., Jones G.R., Tsogtgerel E., Nguyen D., Keyes A., Liu Y.-S., Rauf H., Ordonez E., Puchelle V., Alhan H.B. (2022). Linear block copolymer synthesis. Chem. Rev..

[26] Hadjichristidis N., Iatrou H., Pitsikalis M., Mays J.W. (2006). Macromolecular architectures by living and controlled/living polymerizations. Progr. Polym. Sci..

[27] Dey A., Hldar U., De P. (2021). Block copolymer synthesis by the combination of living cationic polymerization and other polymerization methods. Front. Chem..

[28] Diaz C., Mehrkhodavandi P. (2021). Strategies for the synthesis of block copolymers with biodegradable polyester segments. Polym. Chem..

[29] Theodosopoulos G., Pitsikalis M. (2014). Block Copolymers: Recent Synthetic Routes and Developments. Anionic Polymerization: Principles, Practice, Strength, Consequences and Applications.

[30] Matyjaszewski K. (1996). Cationic polymerization. Mechanisms, Synthesis and Applications.

[31] Kennedy J.P., Iván B. (1992). Designed Polymers by Carbocationic Macromolecular Engineering.

[32] Coessens V., Pintauer T., Matyjaszewski K. (2001). Functional polymers by atom transfer radical polymerization. Progr Polym. Sci..

[33] Hawker C.J., Bosman A.W., Harth E. (2001). New polymer synthesis by nitroxide mediated living radical polymerizations. Chem. Rev..

[34] McCormick C.J., Lowe A.B. (2004). Aqueous RAFT polymerization: Recent developments in synthesis of functional water-soluble (co) polymers with controlled structures. Acc. Chem. Res..

[35] Buchmeiser M.R. (2000). Homogeneous metathesis polymerization by well-defined group VI and group VIII transition-metal alkylidenes: Fundamentals and applications in the preparation of advanced materials. Chem. Rev..

[36] Ofstead E.A., Wagener K.B., Mijs W.J. (1992). New Methods for Polymer Synthesis.

[37] Trnka T.M., Grubbs R.H. (2001). The development of L2X2Ru CHR olefin metathesis catalysts: An organometallic success story. Acc. Chem. Res..

[38] Mortensen K. (2001). PEO-related block copolymer surfactants. Colloids Surf. A.

[39] Adams M.L., Lavasanifar A., Kwon G.S. (2003). Amphiphilic block copolymers for drug delivery. J. Pharm. Sci..

[40] Ulrich K.E., Cannizzaro S.M., Langer R.S., Shakesheff K.M. (1999). Polymeric systems for controlled drug release. Chem. Rev..

[41] Xiong X.Y., Tam K.C., Gan L.H. (2005). Release kinetics of hydrophobic and hydrophilic model drugs from pluronic F127/poly(lactic acid) nanoparticles. J. Control. Release.

[42] Kim S.Y., Ha J.C., Lee Y.M. (2000). Poly(ethylene oxide)-poly(propylene oxide)-poly(ethylene oxide)/poly(ϵ-caprolactone)(PCL) amphiphilic block copolymeric nanospheres: II. Thermo-responsive drug release behaviors. J. Control. Release.

[43] Son K., Ueda M., Taguchi K., Maruyama T., Takeoka S., Ito Y. (2020). Evasion of the accelerated blood clearance phenomenon by polysarcosine coating of liposomes. J. Control. Release.

[44] Hatori Y., Tamaki K., Sakasai S., Ozaki K.J., Onishi H. (2020). Effects of PEG anchors in PEGylated siRNA lipoplexes on in vitro gene-silencing effects and siRNA biodistribution in mice. Mol. Med. Rep..

[45] Kozma G.T., Shimizu T., Ishida T., Szebeni J. (2020). Anti-PEG antibodies: Properties, formation, testing and role in adverse immune reactions to PEGylated nano-biopharmaceuticals. Adv. Drug Deliv. Rev..

[46] De Vrieze J. (2020). Suspicions grow that nanoparticles in Pfizer’s COVID-19 vaccine trigger rare allergic reactions. Science.

[47] Berger M., Toussaint F., Ben Djemaa S., Laloy J., Pendeville H., Evrard B., Jerôme C., Lechanteur A., Mottet D., Debuigne A. (2023). Poly(vinyl pyrrolidone) derivatives as PEG alternatives for stealth, non-toxic and less immunogenic siRNA-containing lipoplex delivery. J. Control. Release.

[48] Torchilin V., Levchenko T., Whiteman K., Yaroslav A., Tsatsakis A., Rizos A., Michailova E., Shtilman M. (2001). Amphiphilic poly-N-vinylpyrrolidone synthesis, properties and liposome surface modification. Biomaterials.

[49] Yamskov L.A., Kuskov A.N., Babievsky K.K., Berezin B.B., Krayukhina N., Samoylova A., Tikhonov V.E., Shtilman M.I. (2008). Novel liposomal forms of antifungal antibiotics modified by amphiphilic polymers. Appl. Biochem. Microbiol..

[50] Liu Y., Luo X., Xu X., Gao N., Liu X. (2017). Preparation, characterization and in vivo pharmacokinetic study of PVP-modified oleanolic acid liposomes. Int. J. Pharm..

[51] Roka N., Kokkorogianni O., Kontoes-Georgoudakis P., Choinopoulos I., Pitsikalis M. (2022). Recent Advances in the Synthesis of Complex Macromolecular Architectures Based on Poly(N-vinyl pyrrolidone) and the RAFT Polymerization Technique. Polymers.

[52] Perrier S. (2017). 50th Anniversary Perspective: RAFT Polymerization—A User Guide. Macromolecules.

[53] Pagidi S., Park H.S., Lee D.Y., Kim M.S., Lee S.H. (2022). Nanosize-confined nematic liquid crystals at slippery interfaces of polymer composites consisting of poly(hexyl methacrylate). J. Mol. Liquids.

[54] Nielsen B.V., Nevell T.G., Barbu E., Smith J.R., Rees G.D., Tsibouklis J. (2011). Multifunctional poly(alkyl methacrylate) films for dental care. Biomed. Mater..

[55] Li J., Barrow D., Howell H., Kalachandra S. (2010). In vitro drug release study of methacrylate polymer blend system: Effect of polymer blend composition, drug loading and solubilizing surfactants on drug release. J. Mater. Sci. Mater. Med..

[56] Heinzmann C., Salz U., Moszner N., Fiore G.L., Weder C. (2015). Supramolecular Cross-Links in Poly(alkyl methacrylate) Copolymers and Their Impact on the Mechanical and Reversible Adhesive Properties. ACS Appl. Mater. Interf..

[57] Khai N., Nguyen H., Dang H.H., Nguyen L.T., Nguyen L.M.T., Truong T.T., Nguyen H.T., Nguyen T.Q., Tran C.D., Nguyen L.-T.T. (2023). Self-healing elastomers from supramolecular random copolymers of 4-vinyl pyridine. Europ. Polym. J..

[58] Oktay B., Baştürk E., Kahraman M.V., Apohan N.K. (2019). Designing Coconut Oil Encapsulated Poly(stearyl methacrylate-co-hydroxylethyl metacrylate) Based Microcapsule for Phase Change Materials. Chem. Select.

[59] Zhang X., Zhang X., Yang B., Wang S., Liu M., Zhang Y., Tao L., Wei Y. (2013). Aggregation-induced emission material based fluorescent organic nanoparticles: Facile PEGylation and cell imaging applications. RSC Adv..

[60] Jennings J., Butler M.F., McLeod M., Csányi E., Ryan A.J., Mykhaylyk O.O. (2018). Stearyl Methacrylate-Based Polymers as Crystal Habit Modifiers for Triacylglycerols. Cryst. Growth Des..

[61] Pingpin Z., Yuanli L., Haiyang Y., Xiaoming C. (2006). Effect of non-ideal mixed solvents on dimensions of poly(N-vinylpyrrolidone) and poly(methyl methacrylate) coils. J. Macromol. Sci. Part B Polym. Phys..

[62] Roka N., Pitsikalis M. (2023). Synthesis and micellization behavior of amphiphilic block copolymers of poly(N-vinyl pyrrolidone) and poly(benzyl methacrylate): Block versus statistical copolymers. Polymers.

[63] Kontoes-Georgoudakis P., Plachouras N.V., Kokkorogianni O., Pitsikalis M. (2024). Amphiphilic block copolymers of poly(N-vinyl pyrrolidone) and poly(isobornyl methacrylate). Synthesis, characterization and micellization behaviour in selective solvents. Eur. Polym. J..

[64] Wan D., Satoh K., Kamigaito M., Okamoto Y. (2005). Xanthate-mediated radical polymerization of N-vinylpyrrolidone in fluoroalcohols for simultaneous control of molecular weight and tacticity. Macromolecules.

[65] Postma A., Davis T.P., Li G., Moad G., O’Shea M.S. (2006). RAFT polymerization with phthalimidomethyl trithiocarbonates or xanthates. On the origin of bimodal molecular weight distributions in living radical polymerization. Macromolecules.

[66] Hadjichristidis N., Iatrou H., Pispas S., Pitsikalis M. (2000). Anionic polymerization: High vacuum techniques. J. Polym. Sci. Part. A Polym. Chem..

[67] Uhrig D., Mays J.W. (2005). Experimental techniques in high-vacuum anionic polymerization. J. Polym. Sci. Part. A Polym. Chem..

[68] Provencher S.W. (1982). CONTIN: A general purpose constrained regularization program for inverting noisy linear algebraic and integral equations. Comput. Phys. Commun..

[69] Roka N., Pitsikalis M. (2018). Statistical copolymers of N-vinylpyrrolidone and benzyl methacrylate via RAFT: Monomer reactivity ratios, thermal properties and kinetics of thermal decomposition. J. Macromol. Sci. Part A Pure Appl. Chem..

[70] Hwang Y., Patterson G.D., Stevens J.R. (1996). Photon correlation spectroscopy of bulk poly(*n*-hexyl methacrylate) near the glass transition. J. Polym. Sci. Part. B Polym. Phys. Ed..

[71] Hempel E., Beiner M., Huth H., Donth E. (2002). Temperature modulated DSC for the multiple glass transition in poly(*n*-alkyl methacrylates). Thermochim. Acta.

[72] Gedde U.W. (1999). Polymer Physics.

[73] Alig I., Jarek M., Hellmann G.P. (1998). Restricted segmental mobility in side-chain crystalline comblike polymers, studied by dielectric relaxation measurements. Macromolecules.

[74] Pitsikalis M., Siakali-Kioulafa E., Hadjichristidis N. (2000). Block Copolymers of Styrene and Stearyl Methacrylate. Synthesis and Micellization Properties in Selective Solvents. Macromolecules.

[75] Ruzette A.-V.G., Banerjee P., Mayes A.M., Pollard M., Russell T.P., Jerome R., Slawecki T., Hjelm R., Thiyagarajan P. (1998). Phase behavior of diblock copolymers between styrene and n-alkyl methacrylates. Macromolecules.

[76] Gikarakis T., Pappas I., Arvanitaki P., Pantazi E., Mitsoni E., Roka N., Pitsikalis M. (2021). Thermal stability and kinetics of thermal decomposition of statistical copolymers of N-vinylpyrrolidone and alkyl methacrylates synthesized via RAFT polymerization. J. Chem..

[77] Moghadamtousi S.Z., Kadir H.A., Hassandarvish P., Tajik H., Abubakar S., Zandi K. (2014). A review on antibacterial, antiviral, and antifungal activity of curcumin. BioMed Res. Int..

[78] Anand P., Kunnumakkara A.B., Newman R.A., Aggrawal B.B. (2007). Bioavailability of curcumin: Problems and promise. Mol. Pharm..

[79] Hewlings S.J., Kalman D.S. (2017). Curcumin: A review of its effects on human health. Foods.

[80] Tomeh M., Hadianamrei R., Zhao X. (2019). A review of curcumin and its derivatives as anticancer agents. Int. J. Mol. Sci..

[81] Scandalis A., Selianitis D., Pispas S. (2021). PnBA-*b*-PNIPAM-*b*-PDMAEMA thermos-responsive triblock terpolymers and their quaternized analogs as gene and drug delivery vectors. Polymers.

