# Supplementary material for: Synthesis, Characterization, and Self-Assembly Behavior of Block Copolymers of N-Vinyl Pyrrolidone with n-Alkyl Methacrylates
Source: Polymers (Basel). 2025 Apr 21;17(8):1122. doi: 10.3390/polym17081122 (PMC12030617; doi:10.3390/polym17081122)
Supplement: Supplementary file 1 [file polymers-17-01122-s001.zip › polymers-3579517-supplementary.pdf]

Block copolymers of N-Vinyl Pyrrolidone with n-Hexyl Methacrylate and Stearyl Methacrylate. Synthesis, characterization, thermal properties and self-assembly behavior in selective solvents

Nikoletta Roka and Marinos Pitsikalis*

Industrial Chemistry Laboratory, Department of Chemistry, National and Kapodistrian University of Athens, Panepistimiopolis Zografou, 15771 Athens Greece

*Correspondence: pitsikalis@chem.uoa.gr

Supporting Information Section

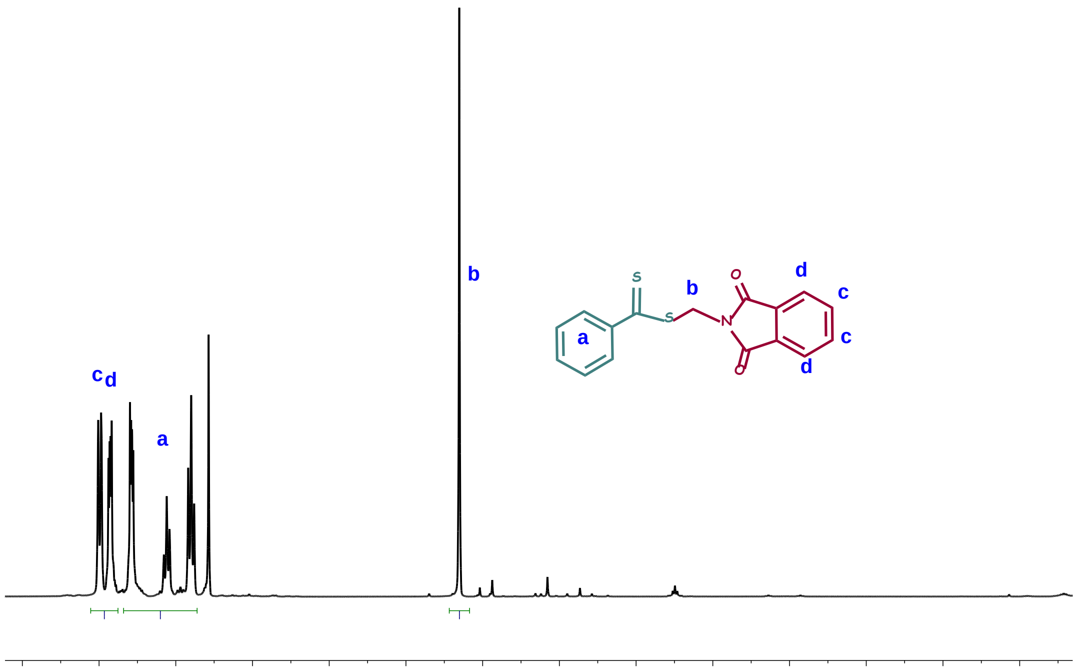

Figure S1. ^{1}H NMR spectrum of CTA MX in $CHCl_3$.

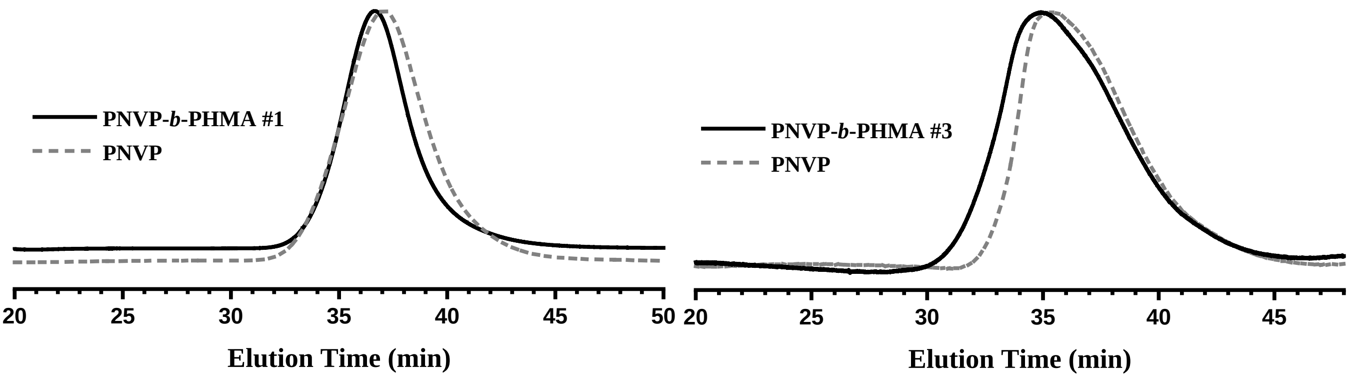

Figure S2. SEC traces of PNVP-*b*-PHMA #1 and #3.

PNVP-*b*-PHMA #1

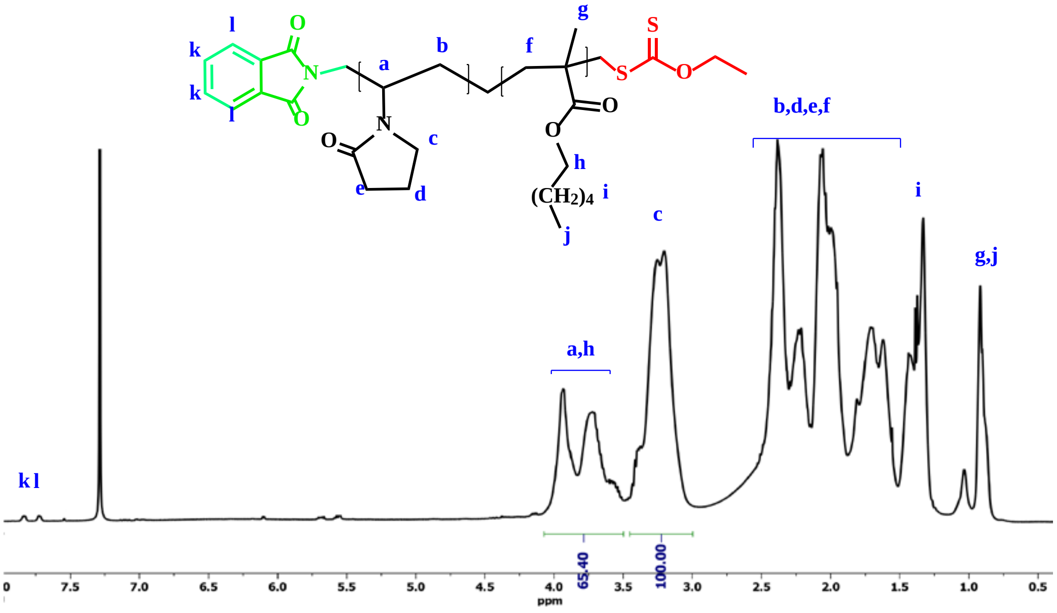

Figure S3. ^{1}H NMR spectrum of PNVP-*b*-PHMA #1 in $CDCl_3$.

PNVP-*b*-PHMA #2

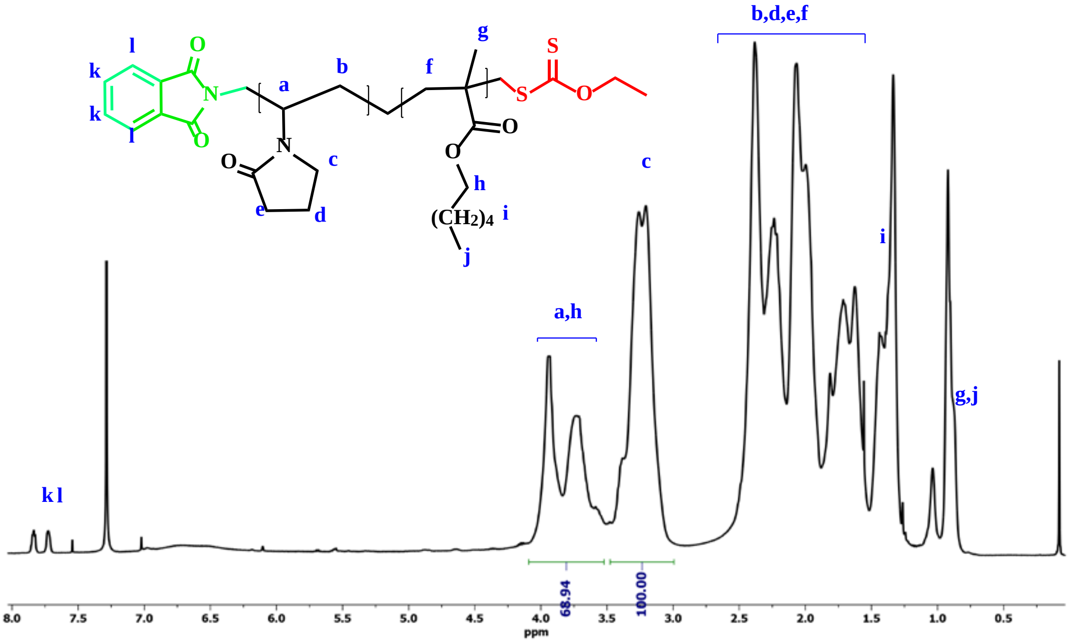

Figure S4. ^{1}H NMR spectrum of PNVP-*b*-PHMA #2 in $CDCl_3$.

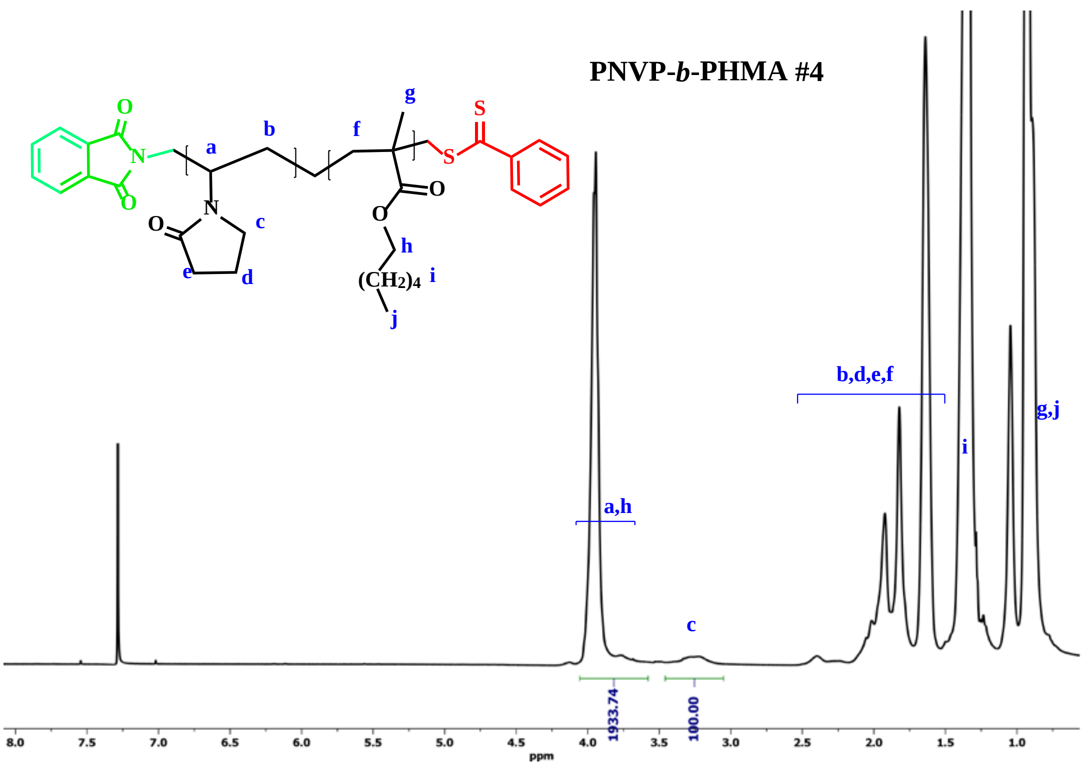

Figure S5. ^{1}H NMR spectrum of PNVP-*b*-PHMA #4 in $CDCl_3$.

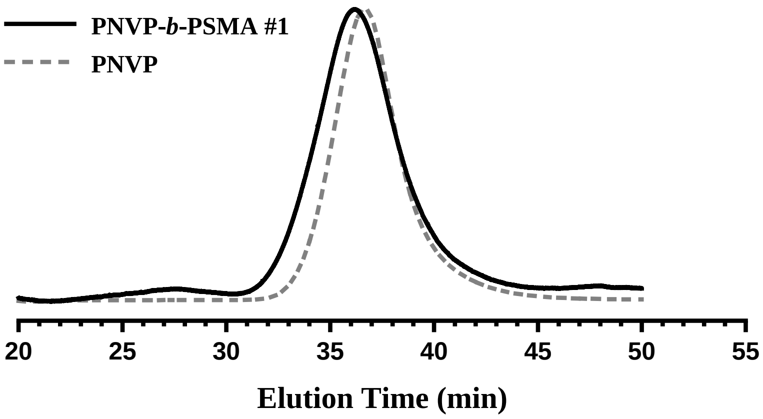

Figure S6. SEC traces of PNVP-*b*-PSMA #1.

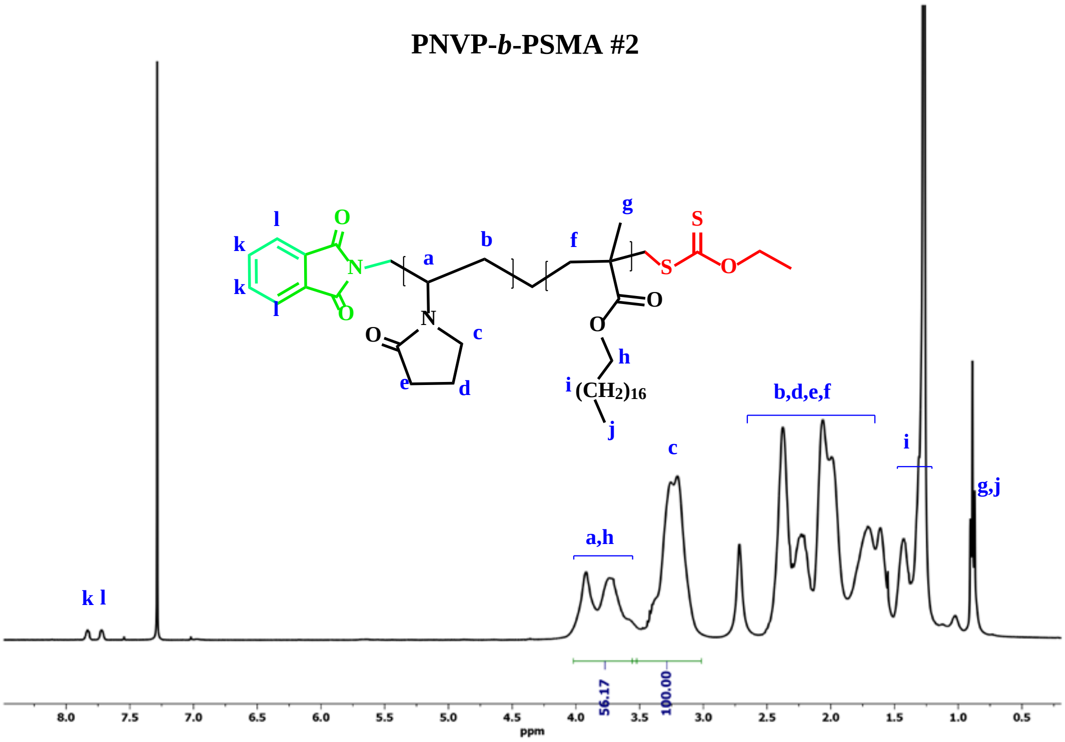

Figure S7. ^{1}H NMR spectrum of PNVP-*b*-PSMA #2 in $CDCl_3$.

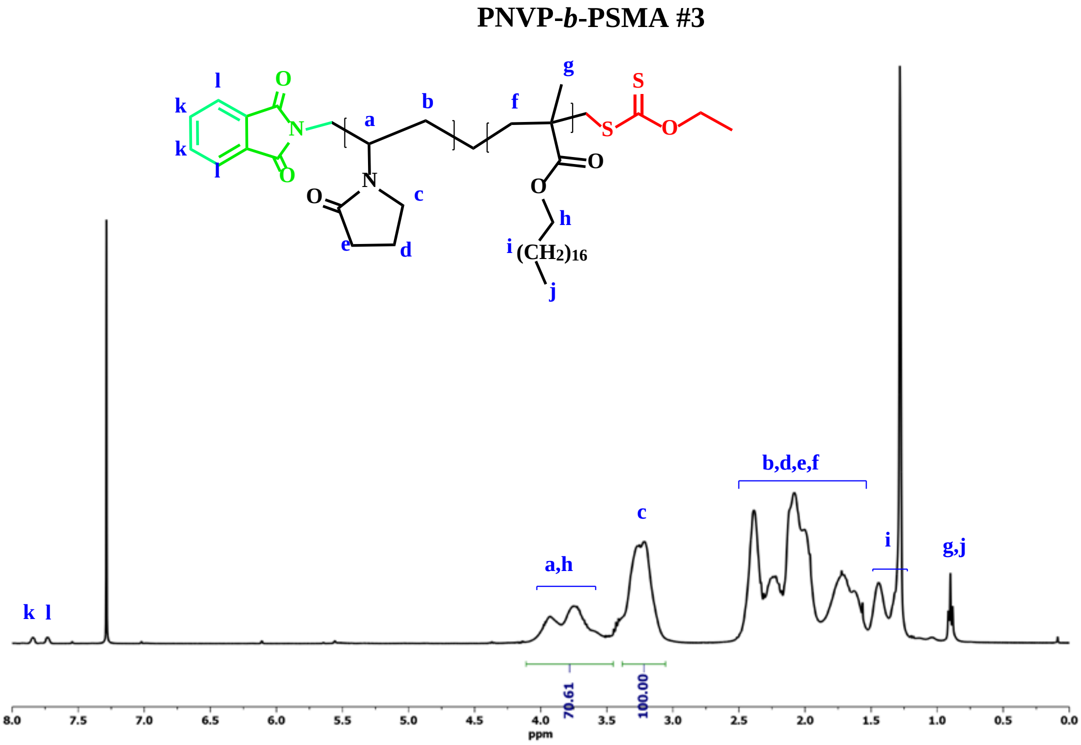

Figure S8. ^{1}H NMR spectrum of PNVP-*b*-PSMA #3 in $CDCl_3$.

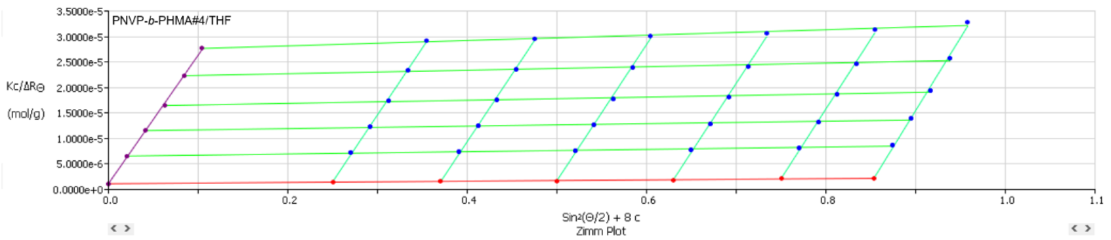

Figure S9. SLS Zimm plot of the sample PNVP-*b*-PHMA#4 in THF

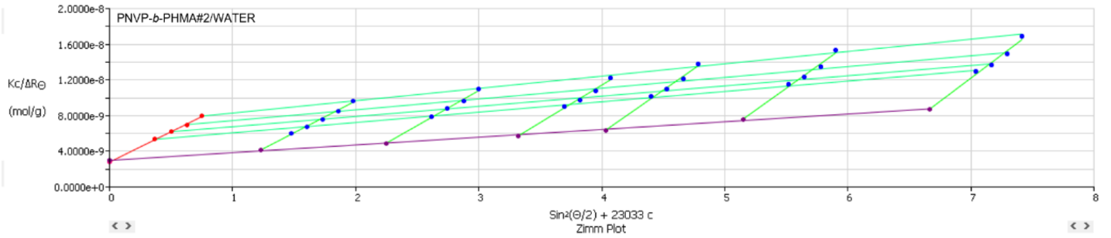

Figure S10. SLS Zimm plot of the sample PNVP-*b*-PHMA#2 in aqueous solution

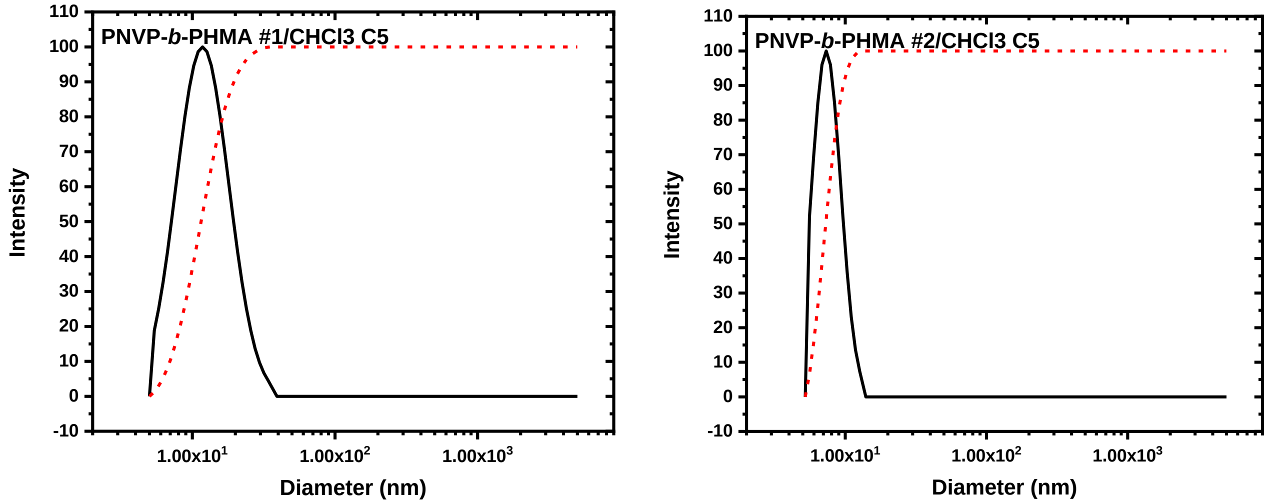

Figure S11. CONTIN plots of PNVP-*b*-PHMA #1 (c=1.400 x10^{-2} mg/ml) and #2 (c=1.983 x10^{-2} mg/ml) in $CHCl_3$.

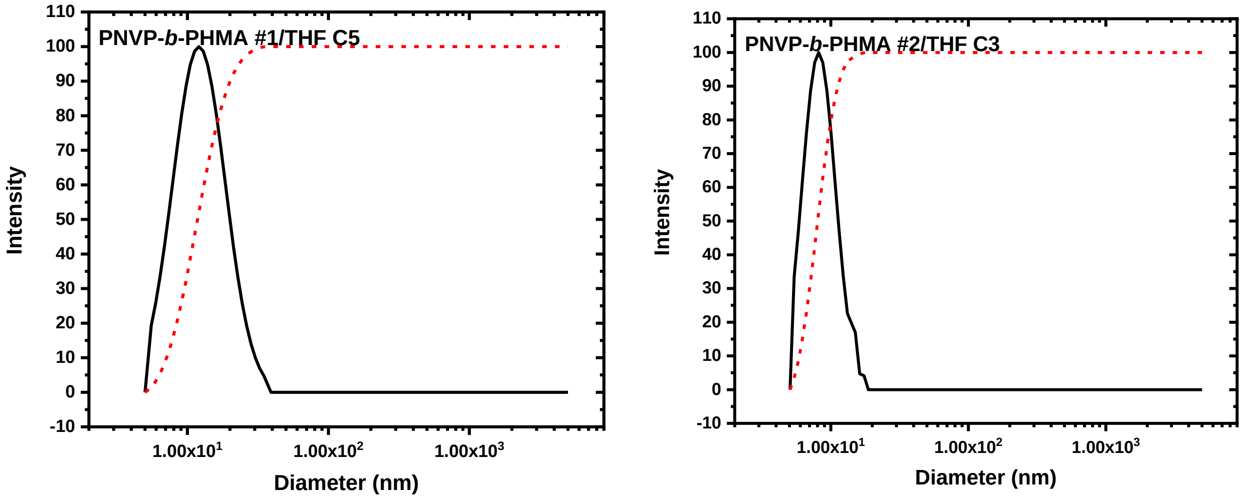

Figure S12. CONTIN plots of PNVP-*b*-PHMA #1 (c=1.330 x10^{-2} mg/ml) and #2 (c=5.665 x10^{-3} mg/ml) in THF.

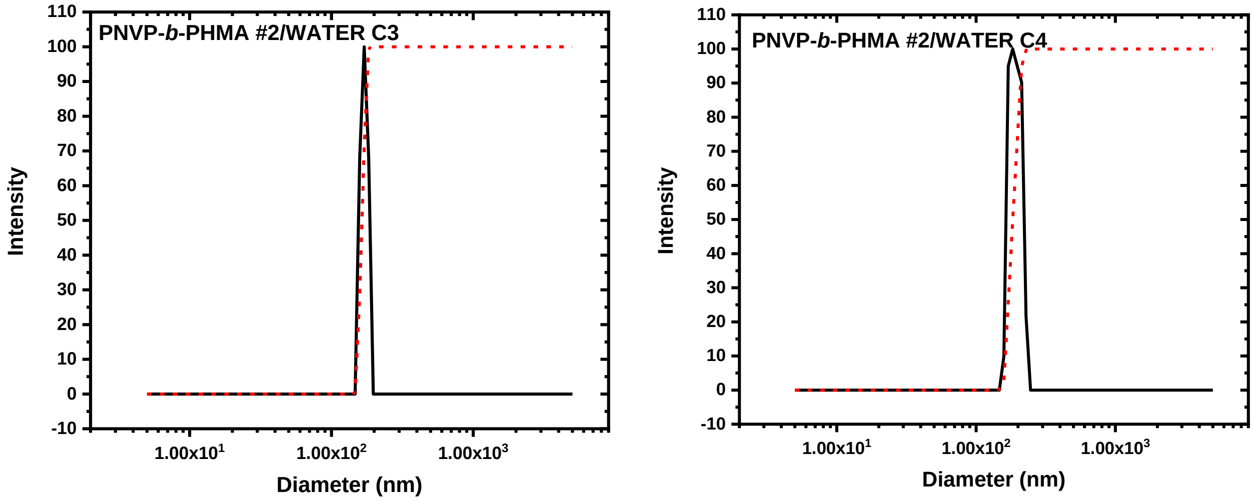

Figure S13. CONTIN plots of PNVP-*b*-PHMA #2 (c3=5.665 x10^{-4} mg/ml and c4=8.196x10^{-4} mg/ml) in water.

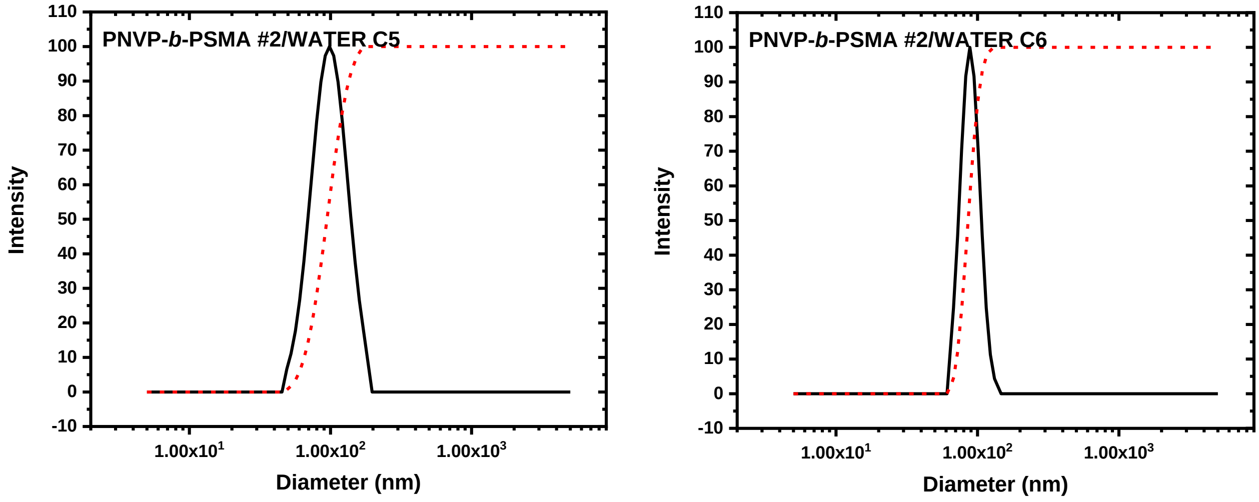

Figure S14. CONTIN plots of PNVP-*b*-PSMA #2 (c5=1.288x10^{-4} mg/ml and c6=1.492x10^{-4} mg/ml) in water.

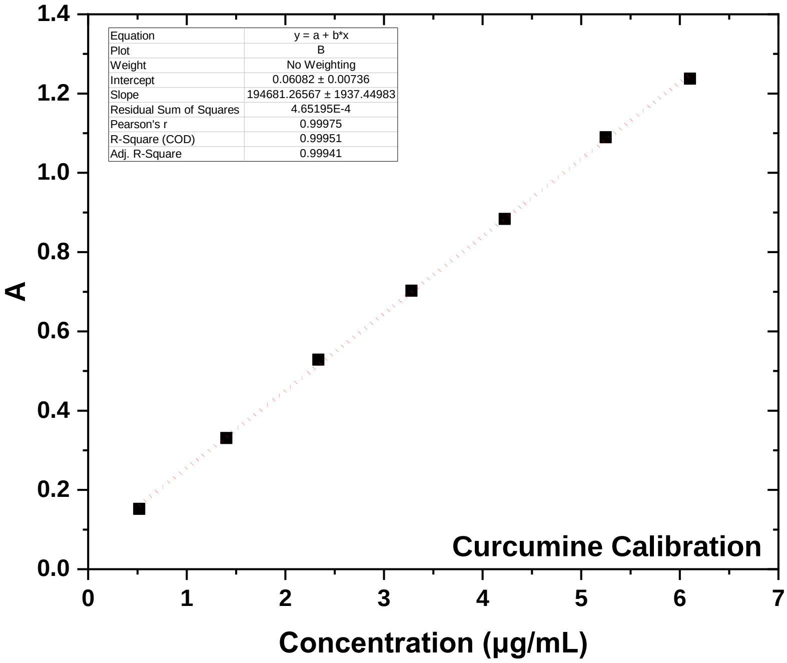

Figure S15. Curcumin calibration curve

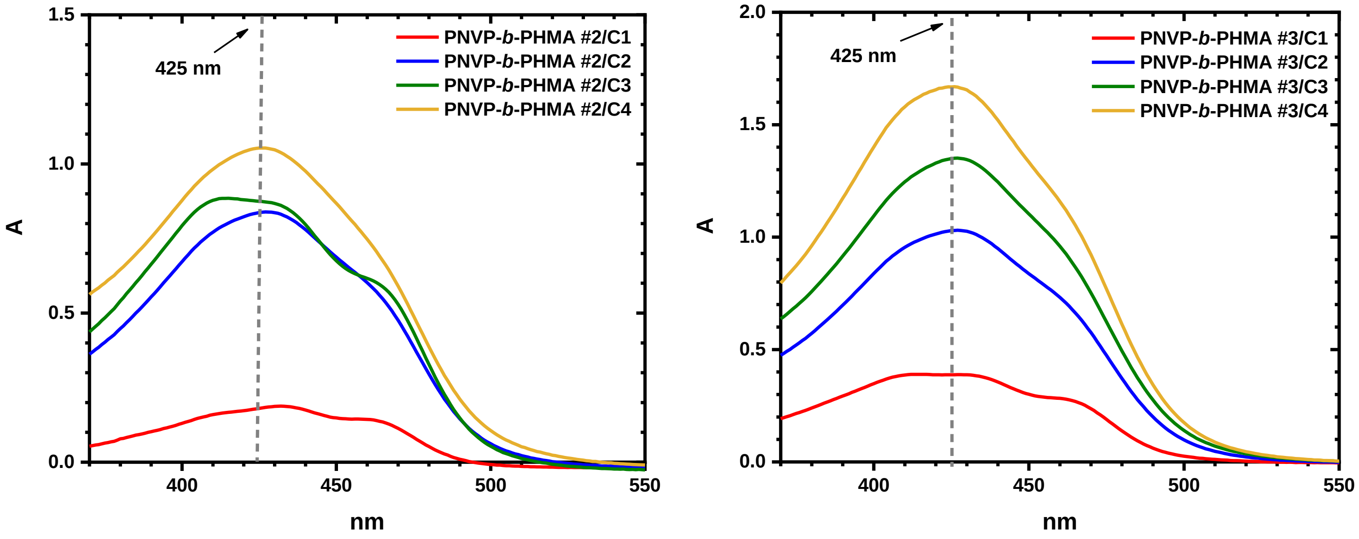

Figure S16. UV-vis spectra for PNVP-*b*-PHMA #2 and #3 with encapsulated curcumin.

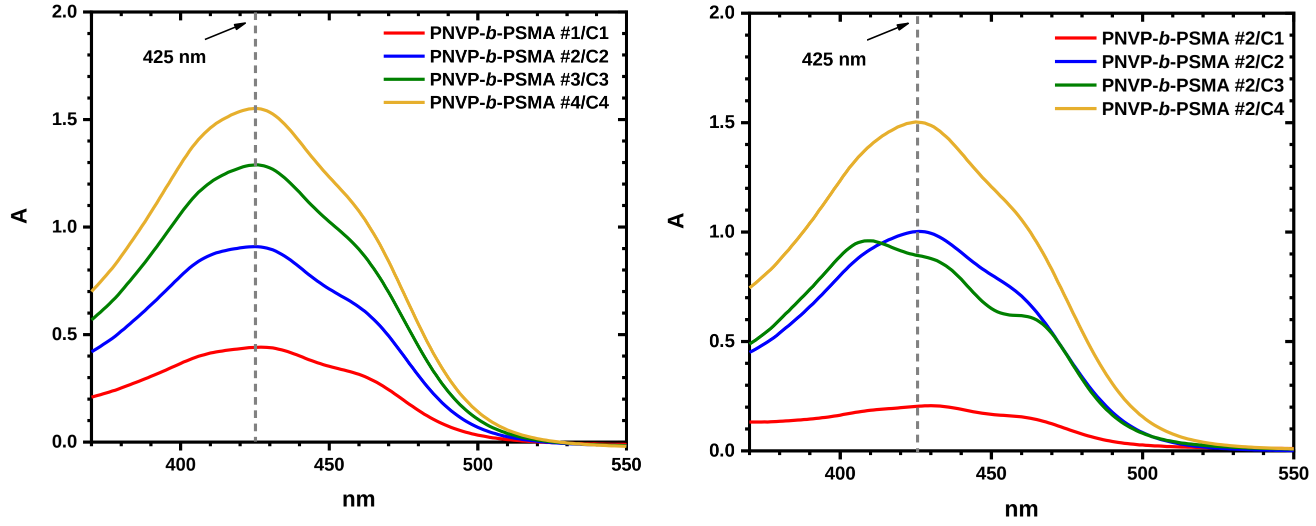

Figure S17. UV-vis spectra for PNVP-*b*-PSMA #1 and #2 with encapsulated curcumin.

Table S1. Quantities for the synthesis of the PNVP-*b*-PHMA block copolymers

Sample	PNVP (g)	AIBN (g)	HMA (mL)	Dioxane (mL)
PNVP-*b*-PHMA #1	5	0.0068	1	9
PNVP-*b*-PHMA #2	5	0.0171	1	9
PNVP-*b*-PHMA #3	1	0.0015	3	2
PNVP-*b*-PHMA #4	0.5	0.0032	3	2

Table S2. Quantities for the synthesis of the PNVP-*b*-PSMA block copolymers

Sample	PNVP (g)	AIBN (g)	SMA (mL)	Dioxane (mL)
PNVP-*b*-PSMA #1	5	0.0068	1	9
PNVP-*b*-PSMA #2	5	0.0178	1	9
PNVP-*b*-PSMA #3	5	0.0178	3	9

Table S3. UV-Vis analysis of PNVP-*b*-PHMA solutions with encapsulated curcumin

Sample	Feed Concentration of polymer	Feed Concentration of curcumin	Concentration of curcumin from UV-Vis
**PNVP-*b*-PHMA #1**	4.8047E-04	-	-
**1/c1**	5.0515E-04	5.8869E-06	2.975013E-06
**1/c2**	5.2300E-04	0.9261E-05	7.084300E-06
**1/c3**	5.2446E-04	1.4807E-05	1.006353E-05
**1/c4**	5.1461E-04	1.9428E-05	7.495229E-06
**PNVP-*b*-PHMA #2**	4.9738E-04	-	-
**2/c1**	5.0134E-04	4.6019E-06	6.121738E-07
**2/c2**	5.0504E-04	1.1050E-05	3.950969E-06
**2/c3**	5.0714E-04	1.4286E-05	4.156433E-06
**2/c4**	5.0526E-04	1.7187E-05	5.081023E-06
**PNVP-*b*-PHMA #3**	4.8593E-04	-	-
**3/c1**	5.1005E-04	4.7316E-06	1.639495E-06
**3/c2**	5.0244E-04	1.0273E-05	4.978291E-06
**3/c3**	5.1545E-04	1.3992E-05	6.622005E-06
**3/c4**	5.1319E-04	1.6952E-05	8.214354E-06

Table S4. UV-Vis analysis of PNVP-*b*-PSMA solutions with encapsulated curcumin

Sample	Feed Concentration of polymer	Feed Concentration of curcumin	Concentration of curcumin from UV-Vis
**PNVP-*b*-PSMA #1**	4.5393E-04		-
**1/c1**	4.8637E-04	4.8732E-06	1.947692E-06
**1/c2**	4.7954E-04	1.0642E-05	4.361898E-06
**1/c3**	4.7458E-04	1.3581E-05	6.262443E-06
**1/c4**	4.5405E-04	1.6272E-05	7.649327E-06
**PNVP-*b*-PSMA #2**	4.9615E-04		
**2/c1**	5.1801E-04	6.0163E-06	7.149059E-07
**2/c2**	5.2216E-04	1.2303E-05	4.824192E-06
**2/c3**	5.2414E-04	1.5926E-05	4.259166E-06
**2/c4**	5.1577E-04	1.8681E-05	7.392496E-06
**PNVP-*b*-PSMA #3**	4.9853E-04		
**4/c1**	5.2942E-04	5.9726E-06	1.434031E-06
**4/c2**	5.2884E-04	1.2247E-05	3.231844E-06
**4/c3**	5.3811E-04	1.5689E-05	6.776103E-06
**4/c4**	5.3665E-04	2.2117E-05	6.005612E-06
